# Review of Potential Drug-Eluting Contact Lens Technologies

**DOI:** 10.3390/ma16103653

**Published:** 2023-05-11

**Authors:** Tina Lovrec-Krstič, Kristjan Orthaber, Uroš Maver, Tomislav Sarenac

**Affiliations:** 1 Community Health Centre Dr. Adolfa Drolca Maribor, Department of Radiology with Centre for Breast Disease, Ulica talcev 5, 2000 Maribor, Slovenia; 2 Department of Anesthesiology, Intensive Care and Pain Therapy, University Medical Center Maribor, Ljubljanska 5, 2000 Maribor, Slovenia; 3 Institute of Biomedical Sciences and Department of Pharmacology, Faculty of Medicine, University of Maribor, Taborska ulica 8, 2000 Maribor, Slovenia; 4 Department of Ophthalmology, University Medical Center Maribor, Ljubljanska 5, 2000 Maribor, Slovenia

**Keywords:** contact lens materials, advanced ocular drug delivery, dropless ocular therapy, drug-laden contact lens

## Abstract

The field of ophthalmology is expanding exponentially, both in terms of diagnostic and therapeutic capabilities, as well as the worldwide increasing incidence of eye-related diseases. Due to an ageing population and climate change, the number of ophthalmic patients will continue to increase, overwhelming healthcare systems and likely leading to under-treatment of chronic eye diseases. Since drops are the mainstay of therapy, clinicians have long emphasised the unmet need for ocular drug delivery. Alternative methods, i.e., with better compliance, stability and longevity of drug delivery, would be preferred. Several approaches and materials are being studied and used to overcome these drawbacks. We believe that drug-loaded contact lenses are among the most promising and are a real step toward dropless ocular therapy, potentially leading to a transformation in clinical ophthalmic practice. In this review, we outline the current role of contact lenses in ocular drug delivery, focusing on materials, drug binding and preparation, concluding with a look at future developments.

## 1. Introduction

Numerous eye conditions, such as chemical or thermal burns, infectious corneal inflammations, optic neuropathies and macular disorders, finally result in very poor visual acuity, thus significantly reducing patients’ quality of life. It is presumed that due to climate change, the incidence of ocular disease might significantly increase, especially in diseases where UV-rays play a role, such as cataracts, age-related macular degeneration and eyelid cancer. Due to population displacement in regard to global warming and rising sea levels, large areas of the globe might end up with worse healthcare, more compromised access to regular examinations and a higher incidence of untreated chronic ocular illness. Poverty-related blindness might increase, on the one hand, while the ageing population in developed countries might further increase the pool of ocular patients. While topical therapy is still the first treatment choice, this method has some major drawbacks. Its efficacy often varies significantly due to subjective factors. Many patients find such an application difficult or even impossible, leading to reduced compliance, which results in poor management of specific eye conditions. Topical delivery media can be irritative and cause dry eye syndrome. Nonetheless, a significant proportion of the drug is drained by the lacrimal system, which connects to the nasal cavity. In recent years there has been an increasing number of examples where various other methods were studied, approaching ocular therapy with a “dropless” idea, addressing the unmet need for a more effective ocular drug-delivery system. 

**Figure 1:**
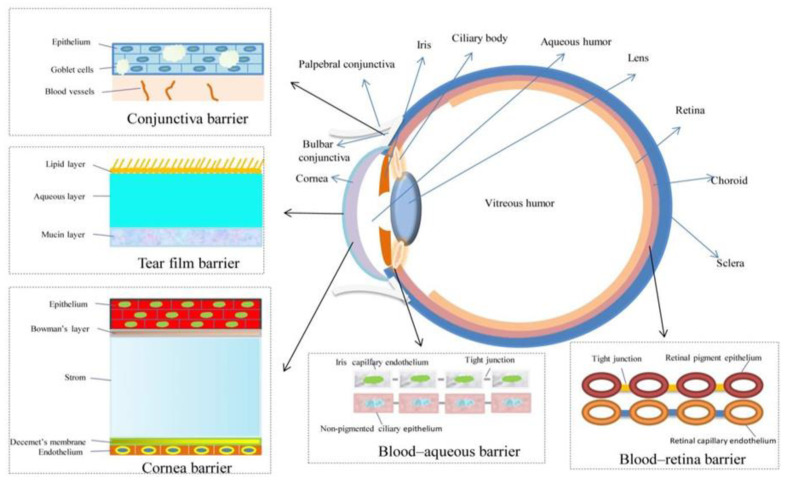
Functional layers and barriers in the eye [2].

Anatomically and physiologically, the eye is also challenging in pharmaceutical terms. Static barriers (cornea, conjunctiva, blood-aqueous humour and blood-retina barriers), dynamic barriers (choroidal and conjunctival blood flow, lymphatic cleansing and dilution due to tear flow) and ocular transporter systems (ATP-binding protein family—P glycoprotein (P-gp) and multidrug resistance proteins (MRP) as well as amino acid and peptide transporters) pose a significant limiting factor in the local drug delivery [1]. A schematical depiction of the eye with its crucial functional features is shown in Figure 1.

Contact lenses are already successfully used for vision correction, while their potential as drug delivery devices is far from fully exploited. We could consider contact lenses as an advanced delivery system for the eye, with a more controlled manner of drug release than common eyedrops, less likely to be affected by poor compliance [3]. While they have been successfully used in past research, we still feel that much can be improved in such devices. Presently, common contact lenses are also used to manage corneal injuries as mechanical protection and to promote healing [4]. They are made of various synthetic polymers, such as silicon and acrylate [5]. 

The current trends in the production of advanced delivery systems are leaning toward natural, biocompatible, smart and potentially cheaper polymers, mostly polysaccharides [6,7]. However, existing contact lenses have an undisputable, albeit clinically underutilised, potential to bind selected substances for advanced forms of topical therapy. Among the most promising methods of drug-binding are colloidal delivery systems (e.g., layered nanoparticles, nano- and micro-myceliums, polymersomes and layer-by-layer (LbL) structures) with the potential to overcome pharmaceutical barriers [2,3]. 

The development of non-invasive delivery systems, such as drug-eluting contact lenses with a prolonged release (of the substance), could result in substantial shifts in the ophthalmological practice, leading to a dropless therapy approach that could simplify therapy for patients and potentially require less follow up by eye professionals. This is more so the case in a pandemic, as we had with COVID-19 when outpatient visits were to be minimised.

**Figure 2:**
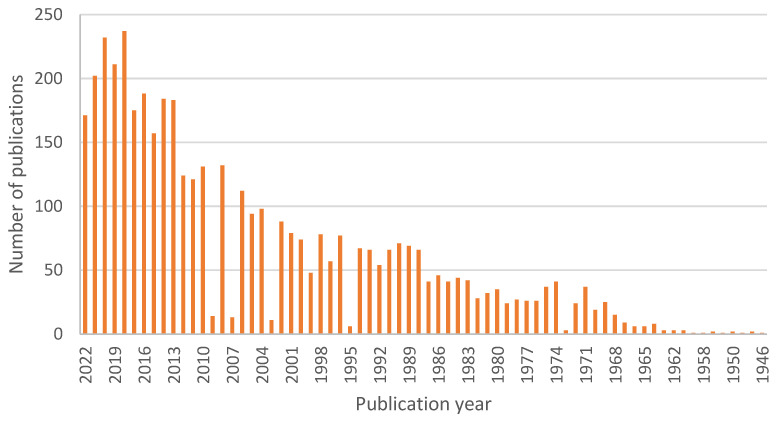
Number of scientific publications by year using a search query “therapeutic contact lenses” done through PubMed on 3 March 2023.

**Figure 3:**
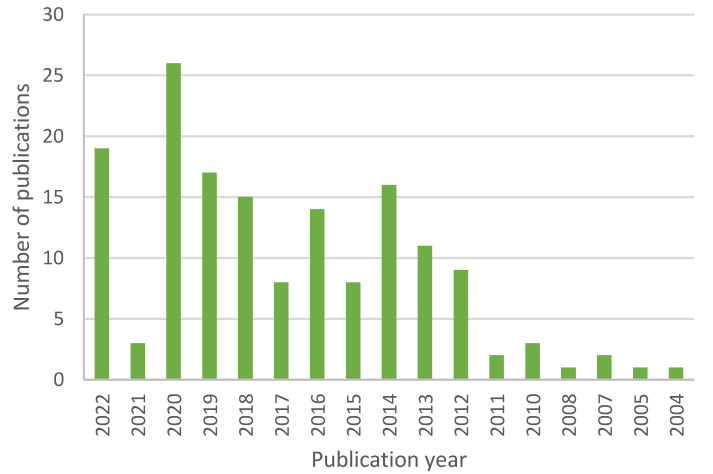
Number of scientific publications by year using a search query “nanoparticle contact lenses” done through PubMed on 3 March 2023.

**Figure 4:**
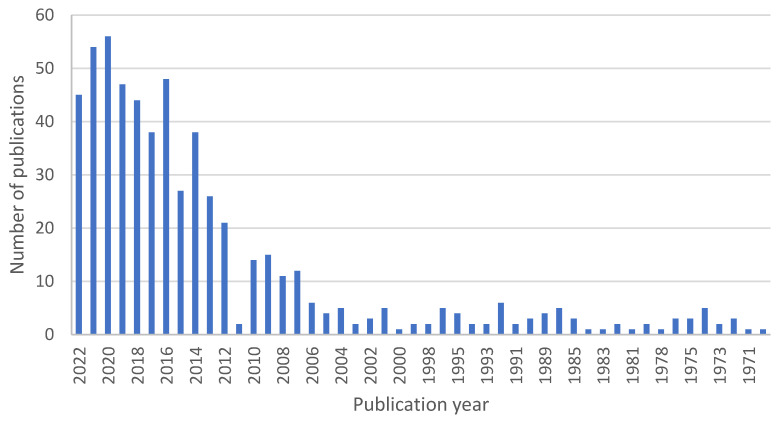
Number of scientific publications by year using a search query “contact lens drug delivery” done through PubMed on 3 March 2023.

For the purpose of this review, we searched through PubMed, where 4011 articles can be found under the search term “therapeutic contact lenses”, 160 for the term “nanoparticle contact lenses”, and 535 for the term “contact lenses drug delivery”. The trend of published articles shows us yearly publication numbers that have been steadily growing, plotted in Figure 2, Figure 3 and Figure 4.

In the following review, we present an overview of the technology currently used in therapeutic contact lenses, as well as several promising possibilities for future applications.

## 2. Lenses

### 2.1. Initial Considerations

Hydrogels, from which most lenses are made, are formed by linking monomeric chains into a matrix-like polymer. Each polymer exhibits unique properties, defined by the chemical group interactions and cross-linking degree [8]. Physical properties are one of the utmost considerations during contact lens design and quality control. Several of these are important when considering a material for use as a drug-laden contact lens system. Among these are their transparency, oxygen permeability, glass transition temperature, wettability and water content. Each of these has to meet certain requirements while simultaneously posing no limitations to the lens use. Transparency, as the lens’s optical clarity measure, must be above 92%. Some approaches can achieve such standards (surfactant-laden SCLs, minimum 98.5% transmittance), while others are somewhat lacking in this respect (liposome-laden Poly(2-hydroxyethyl methacrylate) (pHEMA) gels, 80% transmittance) [9]. Oxygen permeability presents another crucial characteristic of lenses. Its low values can result in unwanted effects, such as corneal oedema. Hence, adequate oxygen transfer is necessary for such systems, with the bare minimum being around 125 Dk/t. It depends on the system’s water content and is therefore limited by its water solubility [8]. Silicone hydrogels are sufficiently capable in this respect, with no substantial alterations in oxygen permeability even soaked in vitamin E to improve drug release times [9]. Wettability is the material property which helps determine the lens’ physiological compatibility and stability in lacrimal fluid and is determined by contact angle measurement. It can be affected by the addition of copolymers [9]. Water content affects comfort and oxygen permeability, which increase with the increase of the property above. It was shown that comonomers do not affect the water content in pHEMA, as is true with molecular imprinting for norfloxacin and fumarate release [9]. Finally, among the lens properties also, glass transition temperature plays an important role. It presents a reversible occurrence in which amorphous material changes from a hard and brittle state into a viscous and rubbery one with increased temperature. Amongst other things, this property also affects comfort. Monomer incorporations do not significantly alter it in pHEMA (compared to pure pHEMA), nor is it affected after the supercritical impregnation process or in the pendant cyclodextrin lenses [9].

### 2.2. Lens Materials

#### 2.2.1. “Classical” Hydrogels

Since poly(methyl methacrylate) was developed in 1928 and commercialised in 1933, polymers have been used in various ways. This material, first discovered during World War 2, is biocompatible with a lack of rejection events after the pilots had suffered Plexiglass^®^ splinter-induced eye trauma [10]. Since 1936, the material has been used in hard contact lenses. However, contact lenses were only popularised in 1954, with the advent of soft pHEMA corrective contact lenses. These polymers have glass-like clarity, which, coupled with lower density and better mechanical properties, made them the beginning of new copolymer lens designs [11]. 

Lens materials are either hydrophilic (which is indicated by the suffix “-filcon”) or hydrophobic (indicated by the suffix “-focon”). These labels are used according to their composition and physical properties. Such classification is mainly used to describe lens behaviour in care product solutions and in interaction with lacrimal fluid proteins [12]. 

**Figure 5:**
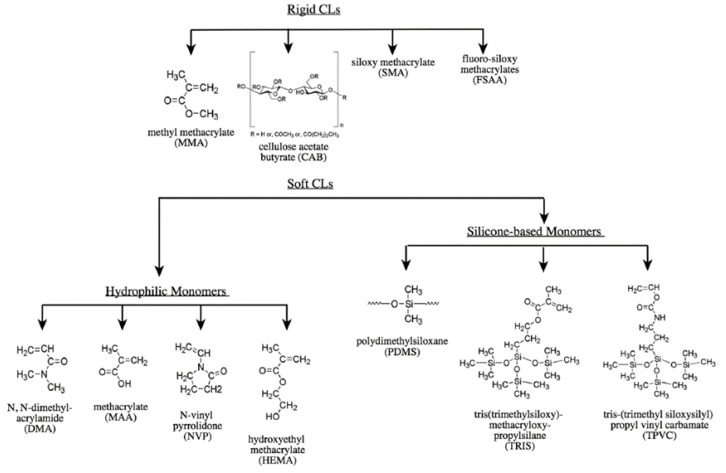
Types of CLs and commonly used monomers [14].

Some of these materials are sufficiently hydrophilic to be used for extended wear. This is largely due to its wettability, enabling sufficient water absorption and retention, which facilitates oxygen absorption and subsequent transport to the cornea [13]. The main types of CL and some commonly used monomers for their preparation are shown in Figure 5.

#### 2.2.2. Silicone Hydrogels

Silicone hydrogels can be worked into a system which prevents hypoxia during closed-eye periods. They make for hard lenses, which are gas permeable, with sufficient optical clarity [11]. 

In general, these materials are strongly hydrophobic. For the contact lenses to work in lieu of this problem, it was necessary to prepare hydrogels that build on a combination of silicone components’ permeability coupled with the biocompatibility of hydrophilic contact lens materials. The result of such a combination is certain contact lenses approved for continuous wear for up to 30 days [15]. 

Since the release profiles of the drug-eluting contact lenses must be fitted to daily, weekly, or monthly disposable products, the time of permeance of the eye surface must remain a critical consideration [13].

Silicone hydrogels have a structure similar to classical hydrogels, with a significantly different chemical composition. Because of silicone’s hydrophobicity, the surface must be modified to improve its incompatibility with the ocular surface, resulting in discomfort. This is due to its poor surface wettability, which destabilises tear film and helps accumulate deposits. The latter can be solved by incorporating soluble polymers within the material, acting as internal wetting agents. These polymers must be oriented to form an interface between the lens, lacrimal fluid, or tear film. This makes them a great combination of a soft contact lens with excellent oxygen solubility in silicone. 

Silicone hydrogel materials are made from a variety of monomers, with the main common property being the presence of silicone. Due to the monomer variety, these hydrogel materials exhibit a differing oxygen permeability, ranging from 60–175 barrers, which is considerably greater than its conventional counterparts. The resulting lenses also vary in stiffness, water content and surface characteristics. The two present hydrogels, FDA-approved for wear up to 30 nights with monthly replacement, are Lotrafilcon A and Balafilcon A. Both are also approved for therapeutic bandage lens uses. Lotrafilcon B is recommended only for daily wear, with up to six nights’ extended wear, while gayfilcon A and senofilcon are only recommended for daily wear [8].

#### 2.2.3. pHEMA

PolyHEMA, with the IUPAC name of poly(2-hydroxyethyl methacrylate), is a soft plastic material used in soft lens production due to its water absorption and flexibility. It is created by polymerising monomer 2-hydroxyethyl methacrylate (HEMA), a clear liquid, a product of a reaction between methacrylic acid and ethylene or propylene oxide.

The contact lens is shaped by casting the HEMA monomer into a small, concave, spinning mould. This is then either heated or subjected to light or free radical initiators, causing the monomer to polymerise, creating long, multiple-unit chains. 

pHEMA chains are usually 3D cross-linked into a complex network by a copolymerising compound. The resulting material is hard but absorbent, meaning it can absorb up to 60% of its weight in water. This results in a soft hydrogel, optically similar to the conventional hydrogels. It is also less irritating to the cornea [16]. 

#### 2.2.4. PLGA

This type of polyester is PLA (poly lactic acid) and PGA (poly glycolic acid) polymer, and it is the best current biomaterial for drug delivery based on its design and performance [17]. Despite its advantageous properties, it is often combined with other polymers. Applying a certain material as a drug delivery device demands an in-depth understanding of the substance’s physical, chemical, and biological properties. With PLGA being a two-part polymer, one must account for both parts’ properties. While the PLA polymer can take either a highly crystalline (PLLA) or a completely amorphous (PDLA) form, with practically no physicochemical property differences, the PGA polymer only takes a highly crystalline form since it lacks methyl side groups [17].

Concerning solubility, PLGA is soluble in various common solvents, not limited to tetrahydrofuran, acetone, and ethyl acetate. It also biodegrades in contact with water since ester linkage tends to hydrolyse. The degradation can be retarded with PLA methyl side groups, which make the material more hydrophobic, with lesser water uptake, resulting in a slower degradation rate. The same degradation process is also responsible for some material parameter alterations, including the change in the glass transition temperature, water content, and molecular weight. All of these changes influence the release and degradation rates of incorporated drugs. Degradation, of course, isn’t the only factor which can influence the material’s properties. Others also include initial molecular weight, device size, water exposure, and storage [18].

The mechanical strength of the polymer, and thereby the device, also depends on various factors, including molecular weight and polydispersity index. The strength is one factor that determines the material’s suitability as a drug delivery device, while it can also influence the degradation rate through the aforementioned hydrolysis. With such contact lenses being drug delivery devices, it is also important to understand that the release rate is not only a function of the lens material but also of the drug used in the formulation [17]. All of the aforementioned properties, namely mechanical strength, swelling behaviour and hydrolysis, depend on the PLGA degree of crystallinity, which also depends on the used copolymer ratio. The decreased crystallinity, namely the fraction of matter in crystalline form, increases the hydration rate and the subsequent hydrolysis. There is a general rule: the higher the PGA content, the faster the degradation process, though the fastest degradation comes with a 50-50 polymer ratio [17]. Crystallinity and the melting point are dependent on molecular weight. Due to its glass transition temperature above 37 °C, the polymers in the body are glassy, with a fairly rigid chain structure [17].

*PLGA composite formulations*

Different drugs require various administration methods for optimal delivery, hence the need for different PLGA combinations with polyethylene glycol (PEG). Production of these copolymers is possible in di- or triblock molecules with different component orders. PEG chains in the diblock types orient themselves towards the aqueous phase, encapsulating their content, and creating a barrier, therefore reducing interactions with foreign molecules. This enhances shelf stability, though it reduces drug and protein encapsulation efficiency [17].

Covalently coupled blocks with an esther link can render triblock copolymers into thermogels: free-flowing solutions at low temperatures and high viscosity gels at body temperature. These copolymers combine the hydrophilicity of PEG segments with the hydrophobicity of PLGA segments to achieve such thermos-responsive characteristics. Drug release from such formulations may be achieved either via diffusion from the hydrogel during the initial phase or through later erosion of the hydrogel matrix [17].

## 3. Methods of Binding Drugs into/to Lenses

The important aspects of drug delivery systems are an appropriate delivery duration, biodistribution, and concentration for the desired therapeutic effect. This means that the design must consider the degradation and clearance of the delivery system and the drug itself. The biodistribution and pharmacokinetics of the polymer in question are dose-dependent and non-linear and also depend on the PLGA system composition [17]. All mentioned is further influenced by various drug incorporation/addition approaches that are considered in the following chapters.

### 3.1. Layered Contact Lenses

Better controlled drug molecule diffusion can be achieved by layering multiple differing materials, enhancing the system’s effectiveness. The layers retard diffusion, ensuring a more stable release. Layering can be achieved in two ways. Firstly, the system can make use of different materials, for one of which the drug exhibits tropism, being less diffusible from that region. Secondly, one can use alternating layers, which degrade over time. Layering methods are numerous, from electrostatic interactions between protein antigens and cationic polymers (rehydration dissolving films) to polymerising surfaces with macromolecules (polymeric structures for the tailoring of drug release) [17]. It is possible to encapsulate a drug-laden PLGA layer inside a PHEMA one, as Ciolino et al. have done, with the PLGA layer being pressed on a photo-polymerised PHEMA layer. The system is then transferred to a mould with a 2-hydroxyethyl methacrylate monomer, which reacts to the top layer, resulting in a film capable of releasing fluorescein in perfect sink conditions for more than four days [19]. This can also be done by taking two identical polymer layers, one of which has a hole, and then fusing them. In the created recess, a low-lens solubility drug is loaded [20].

### 3.2. Surface-Modified Contact Lenses

Drug delivery systems can be created by various methods, relying on loading the drug into “particles” already present in the lens monomer mix during the polymerisation process. The radicals used during the process, however, can cause drug degradation. Such issues can be avoided by loading the drugs into already created polymers or attaching them to the lens surface, enabling us to attach drugs to either side of the lens selectively [21]. Thus, Danion et al. have layered levofloxacin-loaded liposomes on the lens surface, demonstrating that 10 layers of liposomes layered on the lens can achieve a sustained release of six days [17,22]. Qui et al. have invented a method where a positively-charged azetidinuim group is used to affix a hydrophilic and cross-linked polymeric material coating to the lens surface. This can improve the lubricity of the resulting formulation, which may lessen lens wear discomfort [23]. Li et al. have polymerised hydrophilic polymers on the lens surface, catalysing the process with lanthanide or transition metal oxidants [24]. Korogianaki et al. have created a surface-modified lens, covalently affixing Proteoglycan 4 to silicone hydrogel lenses. Surface Proteoglycan 4 addition diminishes the kinetic friction coefficient [25]. A method developed by Winterton utilises coating the lens surface with polyionic materials, creating a wettable surface [17,26].

### 3.3. Soaking Method

**Figure 6:**
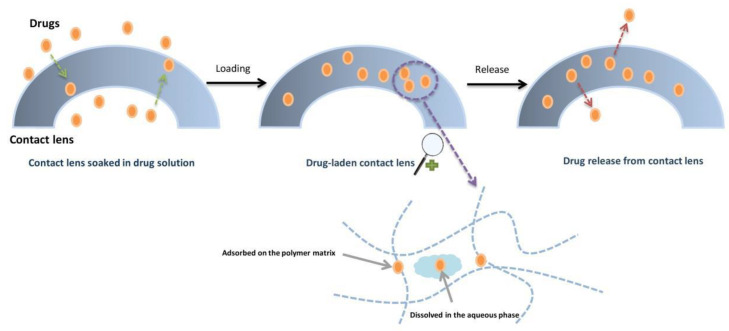
Schematics of the soaking method for drug loading and release [2].

This simplest, cost-effective, and conventional approach to drug loading includes contact lenses being soaked in the desired drug solution. As shown in Figure 6, some of the drug adheres to the polymer matrix while the rest is dissolved in the aqueous phase before release. Drug delivery aspects of the system were investigated in several studies. The tested drugs were (including, but not limited to) timolol [27], dexamethasone, hyaluronic acid and pilocarpine [28,29,30].

Charged surface contact lenses (Balafilcon A, Etafilcon A and B) were shown to have greater ketotifen fumarate uptake as well as release. Their incorporation resulted initially in a burst release, with the fumarate plateauing within a few hours. Compared to eye drop therapy, the contact lenses exceeded the total amount of the incorporated drug [31]. During an evaluation of fumarate-loaded silicone contact lenses, Xu et al. found that the hydrophilic/hydrophobic monomer ratio influenced drug uptake and release kinetics. Increasing the hydrophilic phase of the lens caused a notable increase in drug uptake. Conversely, an increase in the hydrophobic phase meant an improved sustained release. In rabbit eyes, compared to eye drop therapy, a more stable release, lasting 24 h, was noted [32]. A possibility to raise the lens’ loading capacity is by bonding two separate materials, creating a cavity within the lens. However, this compromises O_2_ and CO_2_, as Nakada et al. have found, increasing the risk for corneal oedema [20]. Schultz et al. studied the uptake and release of timolol maleate and brimonidine tartrate using the soaking method to prepare the contact lenses. In vitro studies showed a burst release achieving a plateau in an hour. In glaucoma patients, wearing such lenses 30 min per day for two weeks, the reduction in intraocular pressure (IOP) was equivalent to the previous treatment, even with the drug being diluted ten-fold [33,34]. Using the soaking method, the prepared contact lenses presented a higher drug bioavailability than eye drops [9]. For example, through a combination of in vitro and in vivo studies, Li et al. demonstrated that 20% of the timolol, loaded onto a pHEMA contact lens, entered the cornea, providing a higher fractional uptake in comparison to eye drops [27].

The conventional lens’ ability for drug uptake is highly dependent on the lens’ water content and thickness, as well as the drug’s molecular weight, its concentration in the soaking solution and its solubility in the hydrogel matrix. Soaking time also plays an important role in drug uptake [35]. For example, Maulvi et al. demonstrated that hyaluronic acid, a drug with a high molecular weight, cannot penetrate the hydrogel’s aqueous channels. Thus, it remains on the contact lens surface and even with a higher concentration in the soaking solution, it fails to achieve a corresponding extended release duration [29]. Contact lenses show a low affinity for ophthalmic drugs like timolol maleate, brimonidine tartrate and several others [34,36]. The low affinity causes the drugs to be released in their entirety in a time course of only a few hours. Further, such examples are ketotifen fumarate, ciprofloxacin, prednisolone, acid and pilocarpine [37]. The release characteristics present a further limitation of the soaking method.

While the sterilisation and packaging processes might cause a premature release of the embedded drug, the exact effect has yet to be determined [33]. It was speculated that adjusting the CL loading temperature could optimise the release profile, thereby improving the soaking method. Topoe et al. tested the hypothesis. The CI26Y disks used as intraocular lenses extended drug release to 15 days for moxifloxacin and diclofenac by increasing the loading time to 2 months or the loading temperature to 60 °C. However, the release profile of the Definitive 50 disks, which were used as examples for soft contact lenses, could not be improved by changing the loading conditions [38]. Minami et al. developed contact lenses created with the cast moulding method, which were soaked in epinastine hydrochloride to treat allergic conjunctivitis. Testing out different polymers with varying ionicities in vitro, they discovered that the more anionic polymers showed better release results. Prolonged release was described through in vivo studies on guinea pigs. Also, compared to eye drops, the efficacy of epinastine hydrochloride-soaked CLs was increased in the 12-h study [39].

### 3.4. Molecular Imprinting

**Figure 7:**
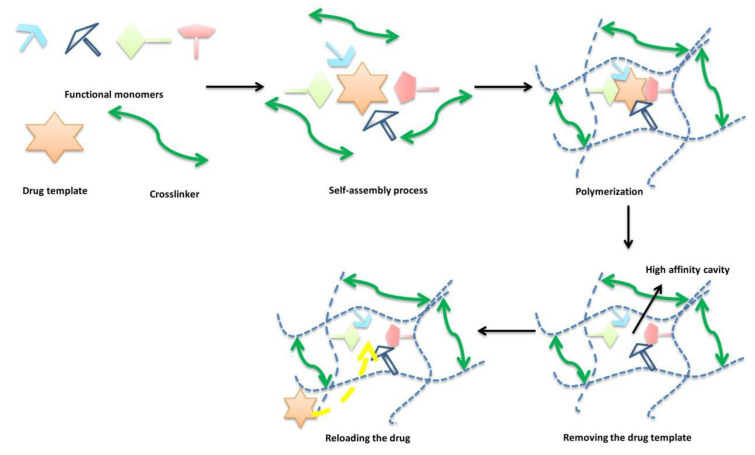
Schematics of the molecular imprinting approach of drug loading [2].

Using a more template-based approach, we can turn to molecular imprinting (MI). With this method, we can achieve the formation of specific cavities in a 3D-polymer network [9,40]. The targeted drug is mixed with functional monomers. All of the molecules are thereby interacting and being rearranged. After polymerisation, the drug is removed. The remaining imprints are essentially tailored active sites, dubbed macromolecular target sites [40]. In other words, the 3D structure of the drug is imprinted onto the inner side of the flexible molecular network. Due to the monomer organisation in the hydrogel mix, molecular sites with high affinity are formed, as depicted in Figure 7, thus increasing the drug loading capacity while decreasing diffusion rates. The macromolecular memory sites also show a high affinity for drugs with a similar 3D structure to the ones used in the manufacturing process. As the drug affinity and its release profiles depend on the type and ratio of the functional monomers in the polymeric matrix, it is possible to tailor them by altering the monomer composition. With an increased degree of cross-linking within the hydrogel matrix, one can attain stable imprinted cavities [33].

MI can be used in conjunction with a variety of monomers, such as HEMA, methacrylic acid (MAA), and methyl methacrylate (MMA; 100–400 mM). The monomers can significantly influence the drug loading capacity, as well as drug release from MI CLs, which was studied by Alvarez-Lorenzo et al. The results showed an increase in timolol loading capacity when MMA was incorporated as a comonomer (100 mM MAA) [41]. Later, Hiratani et al. found that by increasing the MAA/drug ratio, they could achieve a two-fold increase in release duration. The imprinted hydrogels demonstrated a higher affinity for timolol and a slower release rate compared to the non-printed variety [42]. Also, Yanez et al. studied imprinted soft contact lenses for norfloxacin delivery. Their results showed that imprinted hydrogels with norfloxacin/acrylic molar ratios of 1:4 and 1:3 result in the best controllable release process, attaining a sustained release for 24 h [43]. Tieppo et al. have studied the influence of different monomer-to-template ratios and non-ionic covalent interactions. Their research resulted in the development of molecularly imprinted diclofenac-poly(HEMA-co-DEAEM-co-PEG200DMA) soft contact lenses. The latter enabled zero-order release kinetics (in this, the drug is released at a constant rate independent of the dissolved substance’s concentration) at a ratio of 10.5. The release rate in these lenses was almost halved from 11.72 mg/h in the first 48 h [44]. Using multiple functional monomers in MI technology, Vankatesh et al. demonstrated eight times higher drug loading capacity, which poses significant room for treatment individualisation [45]. With the concept being proven in vitro, the next natural step was an in vivo evaluation of CL viability. Hiratani et al. did just that and found that when tested, such lenses with a 34 mg dose of timolol. Compared to non-MI CL with 21 mg timolol and 125 mg applied via eye drops, MI lenses delivered the appropriate drug concentrations 2–3 times longer [46]. Dry eye syndrome is another affliction that could benefit from MI CL usage. White et al. investigated MI silicone hydrogel lenses. They found that release time varies to a notable degree, depending on the functional monomer-to-template ratio, but using 120 kDa Hydroxypropyl methylcellulose, a release of up to 60 days was reported [47].

While various monomers have been shown to favourably influence the loading and release kinetics of the used drug, the MI methodology also has its limitations. For example, the optical and physical properties of CLs can be altered by the structure of such hydrogels. As Schrader et al. would describe it, the chosen template molecules and monomers play an important role in the drug loading capacity, as well as the CL deformation following drug release in their study [48]. The gas and ion permeability is also dependent on the material’s water content, which seems to decrease with the CL wearing time, presenting an obstacle in extended wear uses [49]. In line with these limitations, some authors used other types of structural components for MI. For example, polystyrene can be used to develop a crowding-assisted molecularly-imprinted polymer, as Tang et al. have done. This enabled them to achieve zero-order release kinetics from drug-laden CLs, improving the effective diffusivity [50]. MI was used in the development of polymyxin B-loaded CLs. Malakootin et al. have thus achieved a sustained drug release. The results showed that acrylic acid-functionalised and imprinted hydrogels loaded greater amounts of polymyxin B and had improved sustained release profiles than non-functionalised and non-imprinted networks [51]. Alvarez-Rivera et al. designed epalrestat-loaded hydrogels for the treatment of diabetic eye conditions while also using the method of molecular imprinting. In vivo tests presented a successful accumulation of epalrestat delivered by hydrogels in the cornea. In vitro test results also demonstrated the anti-cataract activity of epalrestat-loaded silicone hydrogels [52]. Anirudhan et al. have discovered a method for achieving an appropriate therapeutic index in sustained drug delivery of timolol maleate with the MI technique [53].

### 3.5. Colloidal Nanoparticles

Colloidal nanoparticles (such as polymeric nanoparticles, liposomes, niosomes, microemulsions, micelles, etc.) can entrap drug molecules and attain better control over their release rate from contact lenses [54,55,56]. Creating therapeutic contact lenses using this approach requires the formulated nanoparticular system (10–100 nm) to be dispersed in different monomers (e.g., HEMA) and further polymerised (e.g., with ethylene glycol-dimethacrylate (EGDMA) and/or with photo inhibitors (e.g., Darocur)) [57,58]. Nanoparticle-loaded contact lenses can provide a controlled drug delivery for an extended time span due to the nanoparticles preventing the interaction of drugs with the polymerisation mixture and providing additional resistance to the drug’s release. Furthermore, enzymatic drug metabolism (such as lysozyme in tears) is also a problem, which seems to be somewhat bypassed using loaded nanoparticles or globules [55,59,60]. We list types and their examples and characteristics of colloidal nanoparticles in Table 1.

Although methods of creating therapeutic CLs are known, we have to keep in mind that any alteration in the CL also poses a risk of altering the lenses’ physical and mechanical properties, such as gas and ion permeability, transparency and swelling behaviour. All of these often had to be changed as well, seeing as the wearer’s comfort has to be considered [33]. The following subchapters review the latest options in therapeutic lenses based on colloidal systems.

#### 3.5.1. Polymeric Nanoparticles

Several researchers have studied polymeric nanoparticles, utilising both biodegradable and non-biodegradable polymers. With the number of ocular pathologies on the rise, such contact lenses were heralded as a novel therapeutic modality. Glaucoma is one such pathology. Jung et al. used dispersed timolol-loaded propoxylated glyceryl triacylate (PGT) nanoparticles in contact lenses. Results of in vitro studies showed a release profile with a drug presence detectable for as long as 1 month. In vivo studies on Beagle dogs demonstrated a reduction in IOP. However, while therapeutically successful, nanoparticles also reduce oxygen and ion permeability in silicone hydrogel CL, as well as increase the storage modulus, thus indicating the technique’s limitation [55]. Timolol encapsulated highly cross-linked nanoparticles, dispersed in CL, as used by Chauhan et al., demonstrated an increase in drug release period to up to four weeks. Monomers with multi-vinyl functionalities like EDGMA and PGT were used to prepare cross-linked nanoparticles. Timolol was linked to the aforementioned CL particle matrix by Esther bonds, the hydrolysis of which is suggested as the drug transport mechanism. While therapeutically worthwhile, the increase in particle bonding also notably leads to an increase in the storage modulus, as well as decreases the water content [61]. Due to its known antimicrobial effects on *P. aeruginosa* and *S. aureus*, silver-impregnated hydrogels were assessed by Bazzaz et al. As it transpires, this method lowers the microbial adverse event risk for extended CL wear [57]. Rad et al. also studied the effects of silver NPs, noting continuous antibacterial activity against *P. aeruginosa* and *S. epidermidis* [72]. Lower needed drug doses are generally desirable, as are decreased dosing frequencies. This was investigated by Chandasana et al. with natamycin in corneal targeting nanoparticle-laden CL. Results of in-vitro studies demonstrated extended release lasting up to 8 h. Compared to marketed preparation, in-vivo studies also presented a notable improvement in the area under the curve and main residence time [73]. Post-cataract endophthalmitis can often present in ocular irritation. Dispersed bovine serum albumin-coated meloxicam nanocrystals in CL material, as developed by Zhang et al., reduced said problem, with a sustained drug delivery lasting up to five days [62].

#### 3.5.2. Cyclodextrins

Due to the physical limitations of hydrophobic drugs in water-rich environments, it is difficult to achieve sufficient doses and sustained delivery. In order to combat both problems, cyclodextrin (CD) utilisation is one of the possible approaches. The hydrophobic interior of CDs can accommodate several hydrophobic molecules, enabling better-controlled drug delivery [63].

Xu et al. used copolymerisation of HEMA with puerarin-β-CD complex to develop pHEMA/β-CD hydrogels. Incorporating the β-CD into hydrogels increases its hydrophilic properties, thereby inducing swelling, as well as increasing tensile strength. This results in an improved drug residence, followed by a higher drug concentration in tears, as well as vitreous humour, compared to conventional eye drops or hydrogel therapy [74]. Fernández et al. aimed to attain sustained acetazolamide delivery to treat glaucoma, which was achieved via drug-poly-CD loaded hydrogel CL, cross-linked with citric acid. This improved drug solubility in CL material, thereby achieving both corneal drug concentration, as well as an extended release lasting several weeks [75].

**Figure 8:**
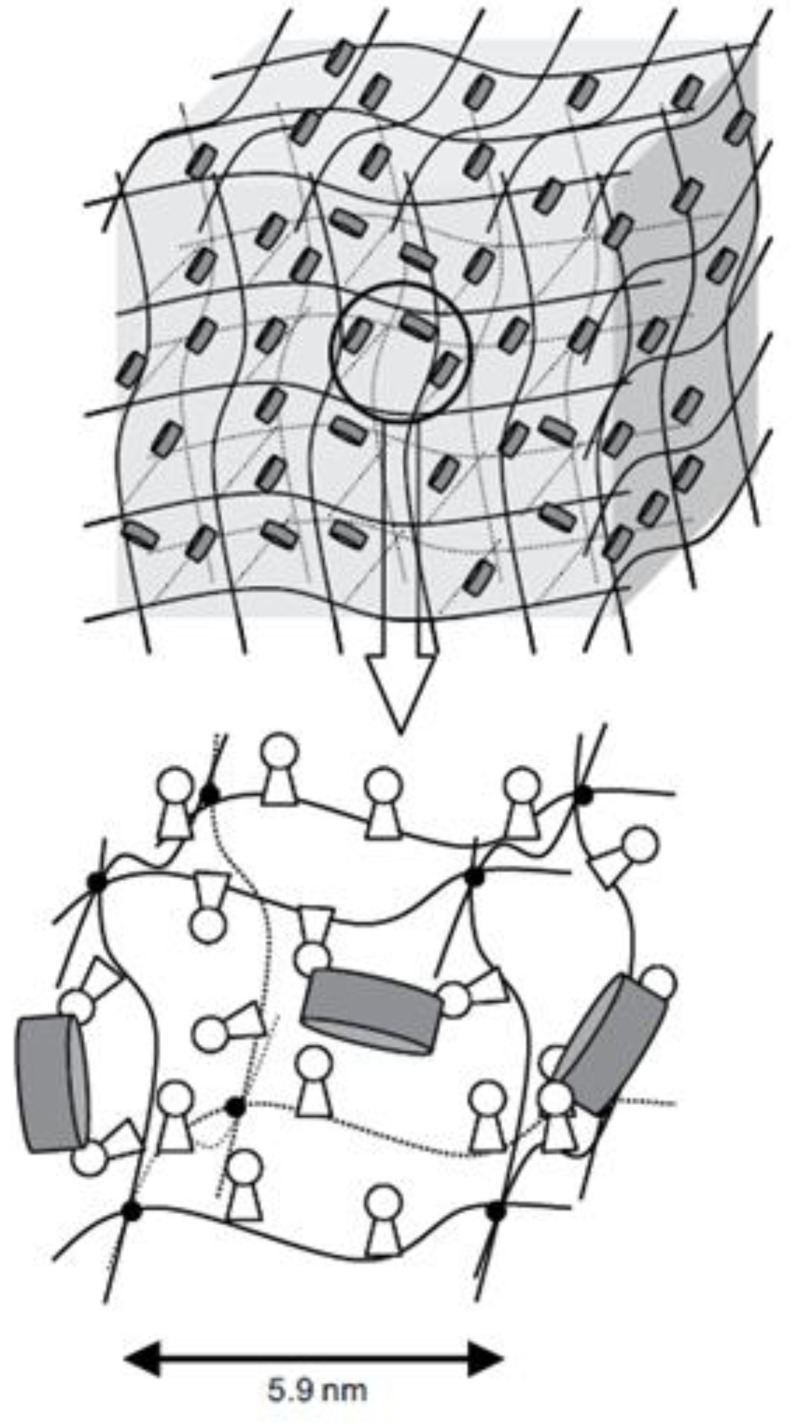
Scheme of a pHEMA-co-GMA hydrogel with pendant bCDs incorporated into its cross-linked network [65].

Copolymerising hydroxyethyl methacrylate with methacrylated-β-CD, as was done by Santos et al., can be used to alter the properties of hydrogel contact lenses, such as drug loading and release rate of acetazolamide and hydrocortisone, by way of alterations in swelling and storage moduli [64]. By copolymerising hydrogels with glycidyl methacrylate (GMA) and grafting them with β-CD, CLs can be adapted for controlled drug delivery. The incorporated β-CDs, bound to glycidyl groups, as shown in Figure 8, did not notably change the hydrogel matrix’s optical and physical properties. This resulted in a 1300% improvement in diclofenac loading, as well as a sustained drug release lasting as much as two weeks [65].

**Figure 9:**
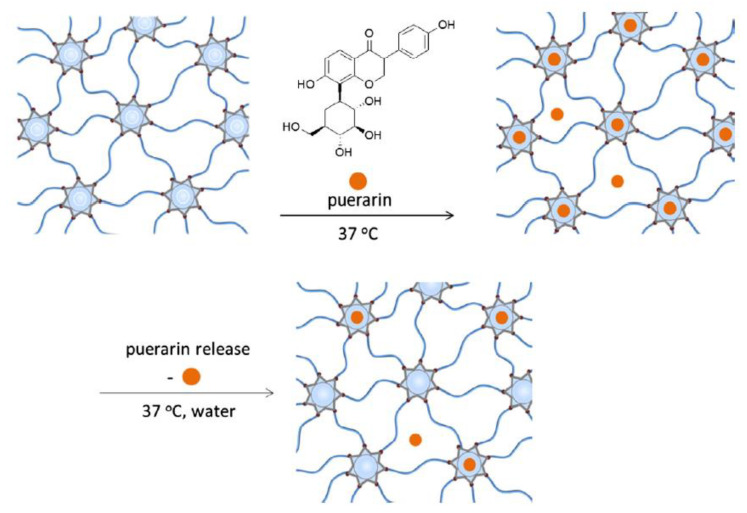
Schematic illustration of puerarins loading and release to CD-containing hydrogels [77].

Glisoni et al. have discovered a way of engineering pHEMA-co-β-CD, which is loaded with an antimicrobial agent (5,6-dimethoxy-1-indanone N-4-allyl thiosemicarbazone). The sustained delivery they achieved lasted up to two weeks though the release profile alters depending on the β-CD proportion, as well as mesh size and the degree of hydrogel swelling [76]. Radical thiolene click chemistry was used by Arslan et al. for the creation of hydrogels using thiol-modified β-CD and allyl terminated PEGs to achieve controlled release of previously loaded puerarins, as illustrated in Figure 9. Similarly to Glisoni et al., they found that altering the amount of β-CD cross-linker or PEG chain length also alters the physical and rheological properties of the hydrogels [77]. Functionalising hydrogels with β-CD can improve the attained ocular drug delivery, as discovered by Hu et al. Further, β-CD functionalised hydrogels demonstrated sustained drug delivery, increased hydrophilicity, and reduced protein adherence [78]. One of the glaucoma treatment options is acetazolamide. By modifying biodegradable polymer CL with β-CD, Prakash et al. achieved a sustained drug release, which was confirmed by observing a prolonged release from the nano-drug complex in simulated tear fluid. It was concluded that the sustained release from the biomaterial shows its great potential in glaucoma therapy since the advantages include fewer side effects of acetazolamide due to a low drug content [79].

#### 3.5.3. Liposomes

By their chemical nature, liposomes create membrane-like structures. These are, due to their biocompatibility and biodegradability, suitable for a variety of medical applications, one of which is being a part of ophthalmic drug delivery CL systems. Liposome-laden hydrogels are a suitable carrier for lidocaine, as Gulsen et al. have shown for dimyristoyl phosphatidylcholine liposomes. In this case, the entrapped lidocaine was released for about eight days. An additional advantage of these liposomes was also the CL transparency [59]. Jain et al. used unilamellar (REL) and multilamellar (ML) liposomes in their drug-eluting CLs. They would load them with ciprofloxacin. The REL variety CLs were prepared via the reverse phase evaporation method, while the ML variety was prepared by the lipid film hydration method. Since there were several lipid barriers, ML liposomes presented a greater sustained release compared to REL liposomes. Moreover, the liposome cholesterol content and liposome structure were observed to play an important role in the amount of loaded and released drugs [66]. Danion et al. immobilised levofloxacin-liposomes on CLs through a multi-step approach. Firstly, the hydroxyl groups on the CL material presented an excellent binding site for polyethylenimine (PEI). Simultaneously, carbodiimide (CDI) chemistry provided an appropriate method of attaching NHS-PEG-biotin molecules to the amine groups. NeutrAvidin was then bound to the PEG-biotin layer in the second step. This resulted in liposomes with PEG-biotinylated lipids that were then bound to the surface-immobilised NeutrAvidin. After adding further layers of NeutrAvidin and liposome layers, multilayers formed [9,21]. A cytotoxicity study has demonstrated that the as-prepared liposome-laden contact lenses are biocompatible [80]. In a related study, Danion et al. have described increasing drug release time with an increase in lipid layers. Namely, CLs with two layers presented up to 30 h of drug release, while 10 layers provided a drug release of up to 120 h [22]. Even though-liposome laden CLs are a promising drug delivery modality, the gas permeability of such systems is compromised due to the liposome multilayer [33].

#### 3.5.4. Microemulsions and Micelles

Using drug-loaded microemulsions and micelle-laden therapeutic contact lenses was shown to be a promising approach to drug entrapment. They are thermodynamically stable and have a high loading capacity for drugs, while their release profile is readily adaptable. Furthermore, their use decreases protein adherence and increases the wettability of contact lenses [67]. Microemulsions and micelles do not seem to obscure the optical traits of hydrogels due to their size (5–100 nm).

**Figure 10:**
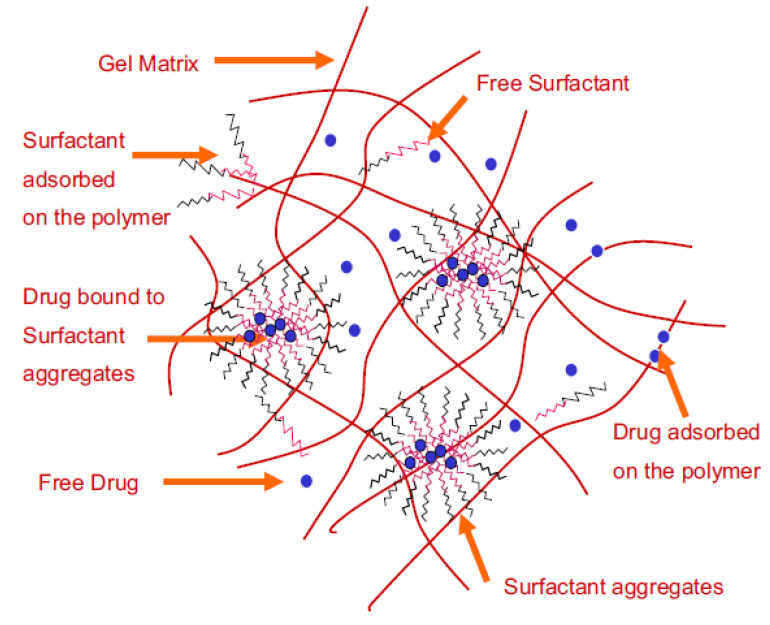
Microstructure of the surfactant loaded gels [82].

Li et al. studied contact lenses for therapy with timolol. They developed lenses with oil-in-water type microemulsions with ethyl butyrate and Pluronic F127. Due to the tightly packed surfactant present at the oil–water interface, the microemulsion demonstrated a controlled release of timolol [68]. A structural schematic in Figure 10 illustrates the interactions between the gel, surfactant and drug.

Bengani et al. developed dexamethasone 21-disodium phosphate-laden contact lenses (ACUVUE^®^, Johnson & Johnson, New Brunswick, NJ, USA), where ionic surfactants were used to increase the affinity of the drug to the charged surface of the contact lens. The procedure resulted in a drug-eluting lens without aberrant optical properties and with less protein absorption. The study demonstrated an increase in the drug release duration from 2 h to 50 h, using loading with 10% surfactant [81]. Gulsen et al. used microemulsion to encapsulate timolol in a formulation stabilised with octadecyltrimethoxysilane (OTMS) silica shell. Such a formulation was later dispersed in hydrogels. The developed hydrogels demonstrated drug release, prolonged to more than eight days. The hydrogel transparency remained unaffected [60]. To extend the delivery of cyclosporine, Kapoor and associates developed nanostructured microemulsions or micelles of surfactant-laden hydrogels. The in-vitro study results presented relatively similar release profiles for micelles and microemulsion-laden hydrogels with parallel surfactant loading. According to the study, the developed hydrogels’ effectiveness was undisturbed by the processing conditions, such as the removal of unreacted monomers (extraction step), sterilisation and packaging [69].

Silica shell cross-linked methoxy (polyethylene glycol)-block-polycaprolactone (MePEG-b-PCL) micelles were used by Lu et al. to load with dexamethasone acetate and fabricate hydrogel contact lenses. The in-vitro study results demonstrated extended release of over a month, while the transparency of the hydrogel material remained unaltered for contact lens wear [70]. Kapoor and Chauhan have shown that a four-fold increase in the loaded surfactant may halve the drug release percentage using their hydrogel drug release model [69]. Chauhan and colleagues developed Barium-laden hydrogels capable of controlled drug release to increase the absorption of cyclosporine. The hydrogels were observed to be effective for cyclosporine and less so for dexamethasone and dexamethasone 21-acetate [82]. The release of ketotifen, a common antihistaminic and MAST-cell stabilizer in ophthalmology, from hydrogel contact lenses was studied by Maulvi et al. Octyltrimethoxysilane was used to formulate silica shell nanoparticles from microemulsion. The in vivo results demonstrated an extended-release of ketotifen of more than 10 days. The silica shell nanoparticles presented good potential as a drug delivery system that does not compromise the critical lens properties [71].

### 3.6. Supercritical Fluid Technology (SCF)

This technology is used to load/impregnate hydrophilic or hydrophobic drugs into contact lenses with supercritical solvents, such as CO_2_. During the impregnation, the dissolution of drugs occurs in a supercritical solvent at conditions that are subcritical or supercritical, followed by an interaction with hydrogels. There are operational parameters which can be adjusted so as to influence the drug’s release kinetics (temperature, time, pressure and depressurisation rate) [33]. Several authors report the use of this technology to successfully prepare novel therapeutic lenses.

Acetazolamide and timolol maleate, both glaucoma drugs, were impregnated to Balafilcon A contact lenses (PureVision, Bausch & Lomb, Laval, QC, Canada) using discontinuous supercritical solvent impregnation with CO_2_ + EtOH and CO_2_ + H_2_O. Costa et al. have further demonstrated that it is possible to attain varied levels of drug impregnation by using variable co-solvent types and compositions, affecting drug loading and the pattern of release, without affecting the optical properties of the lens [83].

Due to the limitations of techniques such as soaking, which can only be used for water-soluble drugs, and molecular imprinting, Yanez and colleagues developed an SCF-assisted molecular imprinting technique to load Hilafilcon B contact lenses (Soflens, Bausch & Lomb, Laval, QC, Canada) with drugs and adjust their release profile. In the case of flurbiprofen impregnation, the sequential SCF increased the loading capacity of the drug. It was suggested that both physical and chemical interactions compel molecularly imprinted cavities since a higher affinity and recognition ability for flurbiprofen in an aqueous solution were noted [84]. In contrast to conventional molecular imprinting methodology, the SCF technique demonstrated a favourable drug release profile, an improvement in drug loading, and unaltered key characteristics of the contact lens [85,86].

In studies by Duarte et al., SCF was employed to molecularly imprint salicylic acid, acetylsalicylic acid and flurbiprofen. In the first two cases, an acrylate (poly(diethylene glycol dimethacrylate)) was synthesised with CO_2_ + carboxylic acid and perfluoropolyether oil as a stabiliser. Results have shown that the drug release rate is influenced by the amount of the drug actually used at the fabrication step and the percentage of its impregnation [87]. In the third case, flurbiprofen was released in a controlled manner for three months. They impregnated the drug into methyl methacrylate-co ethyl hexyl acrylate coethylene glycol dimethacrylate), P(MMA-EHAEGDMA)contact lens matrix [86]. Finally, Yokozaki et al. studied the importance of temperature and pressure in impregnating salicylic acid using supercritical CO_2_. The results demonstrated that low pressure and increased temperature during the impregnation process enable sustained drug release [88].

### 3.7. Vitamin E as a Release-Modifying Additive

**Figure 11:**
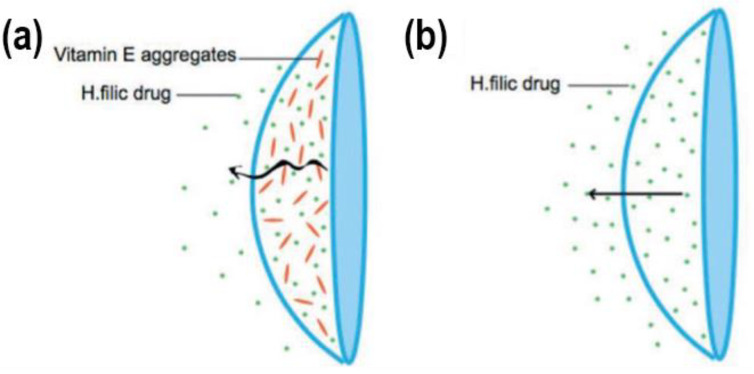
A depiction of hydrophilic drug release from (**a**) vitamin E-loaded contact lens and (**b**) normal contact lens [14].

Diffusion barrier materials should ideally possess low drug affinity to prevent direct diffusion across the barriers. Alternatively, diffusion barriers should notably reduce drug diffusion, even though the drug is trespassing the barriers. Vitamin E presented the best overall performance in slowing the drug release rate without significantly altering the lens’s bulk properties. Vitamin E is very stable when incorporated into a lens due to its hydrophobic nature, having negligible solubility in the tear fluid [17]. In Figure 11, it can be seen how Vitamin E aggregates decrease the rapidity of drug release on a Vitamin E-loaded CL. Several authors used such a strategy to develop their respective drug-delivering lenses.

To increase the duration of drug release, Peng et al. suggested increasing vitamin E loading from 10% to 40%. This has, in effect, created a stable transport barrier for drugs. The results demonstrated prolonged release of timolol. However, oxygen permeability was somewhat hindered [89]. Kim et al. achieved a drug release duration of nine days by developing a dexamethasone contact lens with 30% vitamin E loading. Compared to the drug release time of contact lenses without vitamin E, it was 16 times higher [90]. In a further study by Peng et al., the authors report extended cyclosporine delivery from silicone-hydrogel contact lenses, utilizing vitamin E as the barrier. Sustained drug release of approximately a month was demonstrated in in vitro flux tests using silicone-hydrogel contact lenses. The authors related this change in release to the drug’s high partition coefficient in the gel. In contrast, the hydrogel ACUVUE lens demonstrated release for merely 24 h due to its hydrophilic nature. Thus, silicone-hydrogel contact lenses show immense potential for the delivery of cyclosporine in treating chronic dry eyes [91]. To prolong the release time of cysteamine, Hsu et al. made contact lenses loaded with vitamin E as part of the diffusion barrier. Results demonstrated that incorporating vitamin E improved the release duration from 10 min to 3 h. Not only did vitamin E provide the ability to control the drug release rate, but it also prevented the oxidation of cysteamine [92]. Peng et al. reported another interesting study. Namely, they managed to extend the release duration of charged (at physiologic pH) anaesthetic drugs (such as lidocaine, bupivacaine and tetracaine) to seven days. This was achieved by using nano-sized hydrophobic vitamin E aggregates acting as a diffusion barrier in silicone contact lenses. These therapeutic contact lenses were shown to be a viable possibility for treating post-operative pain that occurred during photorefractive keratectomy surgery [93]. Peng et al. loaded ACUVUE TruEye contact lenses with vitamin E as a diffusion barrier to extend the timolol release duration for the treatment of glaucoma. The results showed a notable reduction in IOP in beagle dogs, using only 20% of the drug dose compared to eye drops therapy [94]. Hsu et al. used a similar approach for two simultaneously incorporated drugs. They achieved sustained drug release of timolol and dorzolamide from a single contact lens to treat glaucoma. Vitamin E was incorporated into the lenses as a physical barrier to sustain drug release [95]. Dixon et al. studied the release of cysteamine using vitamin E-laden contact lenses to treat cystinosis. As cysteamine is very small in size, the incorporation of vitamin E barriers into the lenses prevented the otherwise very rapid release. The results showed an improved release duration of both ACUVUE^®^OASYS^®^ and ACUVUE^®^ TruEye^TM^ [96]. Dixon et al. were involved in another study in which they developed carbon black tinted contact lenses loaded with cysteamine and vitamin E. These lenses were used to achieve cysteamine delivery while also addressing the issue of photophobia that is common in cystinosis. Using pre-made contact lenses resulted in an uneven distribution of carbon black. Therefore, they added 0.3% of carbon black to the monomer solution, creating a lens that still has the required lens parameters for wear and is capable of delivering cysteamine while helping with photophobia. The carbon black influenced the effect of vitamin E, requiring three times the amount of vitamin E loading to achieve the same increase in cysteamine release in comparison to control lenses without carbon black [97]. Sekar et al. explored the release of bimatoprost from polymeric hydrogels incorporated with vitamin E. The following lenses were used: ACUVUE^®^OASYS^®^ and ACUVUE^®^ TruEye^TM^. Loading the lenses with vitamin E enabled the delivery of bimatoprost in therapeutic dosages for over 10 days, prolonging the release duration 10–40-fold, while light transmission and other properties were not compromised by vitamin E loading [98]. Torres-Luna et al. also studied drug release from ACUVUE^®^OASYS^®^ and ACUVUE^®^ TruEye^TM^ lenses. They incorporated the lenses with vitamin E in conjunction with a cationic surfactant. The results demonstrated a sustained release of non-steroidal anti-inflammatory drugs within their therapeutic windows. Diclofenac sodium release was extended to over 150 h for both types of lenses [99].

Vitamin E is also an antioxidant, protecting the cornea from UV radiation and certain drugs from oxidation. Vitamin E showed the ability to extend the release duration of several hydrophilic drugs, showing immense potential as a biocompatible diffusion barrier. Its limitations, however, should not be ignored. For example, vitamin E reduces ion permeability and oxygen permeability and increases storage module, i.e., change in mechanical properties and protein absorption due to its hydrophobic nature [33].

### 3.8. Other Solutions

Hyaluronic acid (HA) can prolong water retention, slow tear removal, improve tear film stability, decrease protein adsorption at the ocular surface and allows undisturbed blinking [100]. It is usually found in eyedrops or artificial tears. This means that binding it to the surface of a contact lens could prolong these effects.

Singh et al. investigated contact lenses modified with a hyaluronic acid-binding peptide (HABpep). Their ability to locally bind and concentrate hyaluronic acid, applied with eyedrops, made for better water retention. They covalently HABpep-modified the contact lens surface with and without a poly(ethylene glycol) (PEG) spacer to bind HA noncovalently. The fluorescence measurements showed increased water retention in HABpep-modified lenses with or without a PEG spacer. It is assumed that HABpep-modified contact lenses could localise HA applied via eyedrops or contact lens solution in a thin coating, biologically and physically benefitting the ocular surface [101].

Table 2 summarises all the drug-binding methods to contact lenses described above, with their advantages and limitations.

## 4. Contact Lenses Preparation Methods for Use in Drug-Delivery

**Figure 12:**
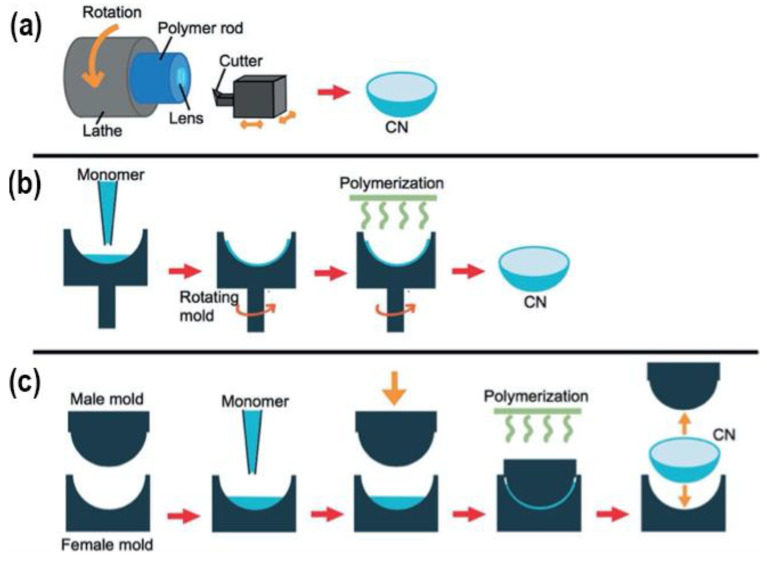
Preparation methods of CLs: (**a**) lathe-cut, (**b**) spin casting, and (**c**) injection moulding [14].

Therapeutic contact lenses can be prepared through various methods. Each of these has its advantages and disadvantages, affecting their respective use for targeted drugs and/or applications. Here some of the most commonly used methods for this purpose are reviewed and displayed in Figure 12.

### 4.1. Cast-Moulding Method

In the cast-moulding method, the lenses are manufactured by depositing a curable mixture of polymerisable monomers into a mould cavity. The mould cavity is formed with two mould sections. The anterior mould section forms the anterior lens surface, while the posterior mould section forms the posterior lens surface. After curing the monomer mixture, the mould is disassembled, and the lens is removed. This method is often used for manufacturing disposable contact lenses due to the quick polymerisation process. The rapid polymerisation times tend to produce less efficient cross-links with shorter chains and more chain ends. The presence of oxygen during the manufacturing process could result in degeneration of the lens surface. Since cast-moulded lenses have a low volume-to-surface area ratio, oxygen degeneration could affect the polymer network [102].

Maulvi and colleagues used a modified cast moulding technology to embed the drug-laden rings into the contact lenses, thus creating an efficient dual drug delivery system. They created a novel type of lens containing semi-circular acrylate rings laden with moxifloxacin HCl and hyaluronic acid. In vitro, studies demonstrated a release of moxifloxacin and hyaluronic acid of up to 96 h. In the in vivo studies, the single implant contact lens therapy showed a healing effect comparable to high-dose eye drop therapy [103].

### 4.2. Lathe-Cutting Method

This method creates lenses from a cylindrical button of dehydrated material. It starts with injecting the polymer material into a glass tube, which is then heat treated. The created polymer rod is then cut to produce a cylindrical button, which is then shaped in the lathe. The frequent use of thermal initiators possessing low activation energies enables the use of relatively low temperatures in ovens or water baths [2,102]. Similar to the cast-moulding method, oxygen could cause surface degeneration of the polymer, but due to the higher volume-to-surface area ratio, it presents less of a problem. The lens can simply be formed from the centre of the button, and the degenerated surface is discarded [104]. In this method, the polymer structures tend to have longer chains, also meaning more chain entanglements. Furthermore, the lenses are commonly heat-treated, causing more possible changes in their mechanical characteristics [105].

### 4.3. Spin-Casting Method

In this lens manufacturing process, the monomer mixture is injected into a rotating mould and spun at the desired speed by a computer-guided system. The anterior surface of the lens is shaped by the mould. The posterior surface is defined by several factors: the centrifugal force caused by the rotation of the mould, gravity, as well as friction forces and surface tension between the polymer and mould. The lens’s posterior surface geometry and dioptric power can be altered by changing the spin speed [102]. This method is rather quick in contrast to the lathe-cutting method, as the final lens is mostly polymerised within 60 min or less. The mechanical properties can be changed by integrating a diluent into the monomer mixture, aiding in a better polymerisation result [106]. The diluent provides improved access to the growing polymer chains, eases the process of removing the lenses from the moulds, and presents a possible way of controlling the material’s swell factor. Similar to cast-moulded lenses, spun-cast lenses also have a low volume-to-surface area ratio, which means oxygen could cause surface degradation. Therefore, the process is commonly executed under anaerobic conditions [102].

## 5. Future Prospects

While therapeutic contact lenses have seen some advances, loading drugs onto contact lens surfaces remains challenging. Various drugs might require different binding methods and can alter materials’ mechanical properties. For example, vitamin E as a barrier agent in certain drug-laden contact lenses is hydrophobic, which causes protein adsorption, decreased transparency, and increased risk of keratitis. This could be somehow amended by using less-absorptive copolymers in the structure to yield a more hydrophilic quality of the lens [5]. Different polymers have also been shown to work well with nanoparticles, offering a high-loading capability. For instance, Jung et al. incorporated propoxylated glyceryl triacrylate (PGT) nanoparticles carrying timolol into silicone hydrogels, achieving continued drug release over a period of one month. They also demonstrated the incorporation of timolol-laden PGT nanoparticles onto commercial lenses. Further studies showed that such an approach is suitable for in vivo studies but is not viable for commercial use, as the drug is released from the nanoparticles during storage into the gel and packaging medium [55]. The copolymerisation of cyclodextrins with monomers such as HEMA or EGDMA has been researched for the sustained release of hydrophobic drugs. The drug release in this study was often extended to several weeks [65,75]. While methods using nanoparticles provide extended and sustained drug release, some drawbacks must be considered. For instance, PGT nanoparticles have lower ion and oxygen permeability, and a decrease in water content has also been observed [55,61]. On the contrary, it was found that β-CDs have no notable impact on the optical and physical properties of hydrogels [65]. Furthermore, Maulvi and associates developed silica shell nanoparticle-laden hydrogel contact lenses to deliver ketotifen that did not alter the lens’s critical properties [71]. Another promising method here would be molecular imprinting, with different advantages. Molecular imprinting studies have shown that using a combination of HEMA with methacrylic acid (MAA) and methyl methacrylate (MMA), monomers can significantly increase loading capacity and attain a sustained release of drugs such as timolol [41,42]. While macromolecular memory sites and higher cross-linking are an excellent way to increase drug affinity, it also presents a limitation as it makes the method depends on the template molecules and functional monomers. Similar to nanoparticle binding methods, the drawbacks include decreased water content with lower ion and oxygen permeability because of the higher cross-linking [33,48].

**Figure 13:**
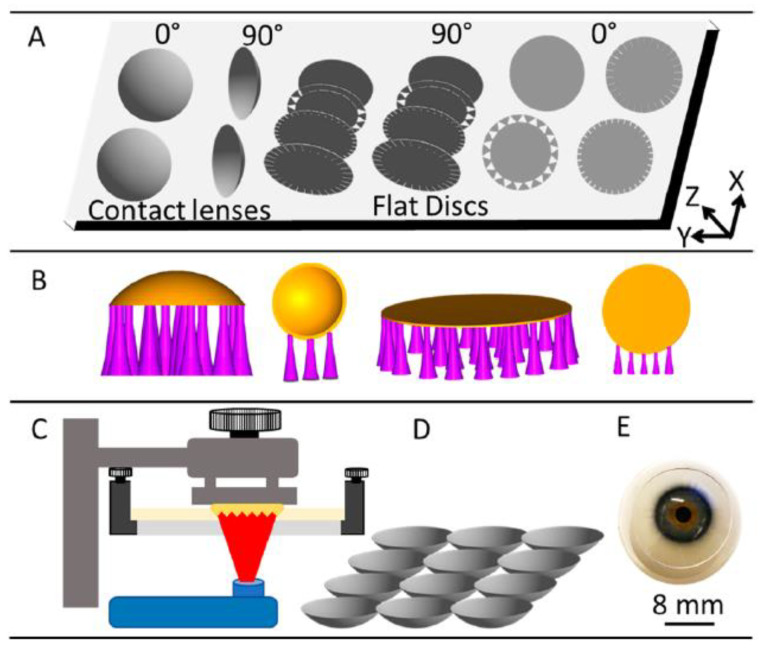
A schematic of contact lens production using 3D printing. (**A**) Computer-designing a model of the lens, (**B**) writing 3D printer readable files, (**C**) digital light 3D printing, (**D**) cleaning and storage of the prepared lenses, and (**E**) patient application of the contact lenses [114].

With the advent of increasingly customisable treatment options in ophthalmology, it would be advantageous to produce different therapeutic lenses for different illnesses. However, lack of generality means questionable real-life clinical applicability of the pharmaco-therapeutic contact lenses. This could change with the advent of customised production methods. One such method is 3D printing, which is becoming widespread in various fields of biomedical applications. In Figure 13 the process of preparation and 3D printing is summarised. While this idea does seem promising, there has been a lack of relevant research in the field. In our previous studies, we have shown that 3D printing can be used to optimise the mechanical properties, biocompatibility and other properties of various formulations [107,108,109,110], which can be transferred to the preparation of novel lenses. In this sense, there are also a lot of opportunities to transfer this knowledge to ophthalmology and prepare therapeutic lenses with desired transparency, mechanical properties, resisting tear and wear, and providing personalised drug delivery tailored to specific patient/disease needs. All in all, this approach might be a valid option for patient-to-patient customisation needed in certain cases.

There are already some related reports found in the literature and a review on 3D printing in ophthalmology that mentions contact lenses as a potential printable drug-delivery system [111]. For example, Fahad et al. have studied the feasibility of 3D printing contact lenses. They used commercially available transparent resin monomers in the printing process, creating discs that achieved a 90% light transmittance. Moreover, they printed tinted lenses for the potential treatment of colour blindness. They also studied the possibility of lenses containing microchannels acting as a transducer for sensing ocular parameters. A 3D-printed drug-eluting contact lens with a central aperture was produced using fused deposition modelling, where printable filaments of copolymers (ethylene-vinyl acetate and polylactic acid) were used. These were beforehand loaded with timolol maleate and have shown a favourable sustained release profile [112]. However, it was shown that digital light printing (DLP) is preferred in contact lens printing due to its ability to achieve a significantly higher resolution than fused deposition modelling [113]. Nevertheless, further research is needed to assess such lenses’ physical and optical properties [114].

In the field of ophthalmic therapy, research has been increasingly invested in finding suitable stem cell therapies. It has already been shown that stem cells can migrate from a carrying medium to the ocular surface, demonstrated by a novel product in regenerative medicine of ocular surface—Holoclar, approved by the European Medicines Agency (EMA). A possible route to future development of advanced medicinal products for the ocular surface could very well be a 3D-bioprinted contact lens carrying stem cells. However, more research into this area needs to be done, as in the paper of Gruene et al., where they have shown that stem cells could be successfully 3D-printed into a material using laser-assisted bioprinting to generate 3D grid-shaped grafts of human adipose-derived stem cells and a natural hydrogel (alginate-blood plasma compound) [115].

**Figure 14:**
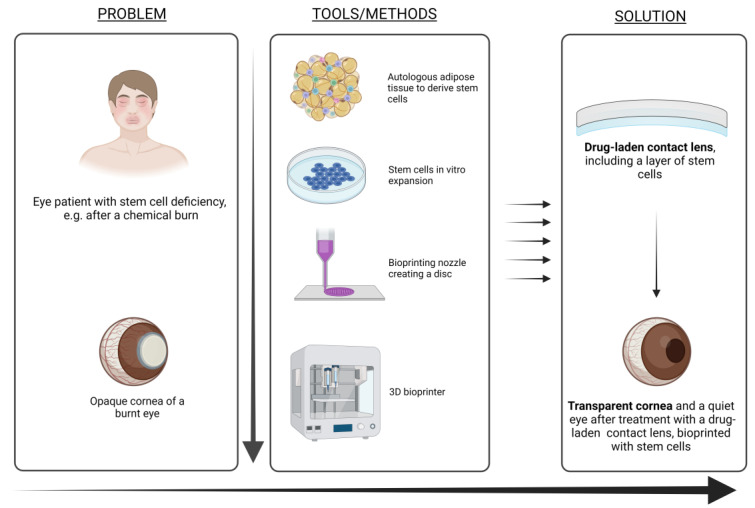
A scheme of possible future use of contact lenses for ophthalmic therapy. In this case, limbal epithelial stem cells’ deficiency in, e.g., chemical burn patients is considered an exemplary problem. With the help of 3D bioprinting, stem-cell-laden contact lens formulations can be printed in desired forms and geometries. If necessary, the same formulation can be upgraded with additional pharmacotherapeutics. Such an approach might present the future of one-step dropless individually tailored therapeutic options in ophthalmic ocular therapy. With related tools that are being continuously developed and interdisciplinary technical solutions, the future prospects of contact lenses in therapeutic use seem promising.

A very limited number of review articles on the topic of therapeutic contact lenses have been published to date [2,3,4]. While these predominantly focused on contact lenses and drug delivery vehicles, our review is more focused on a concise review of the latest novelties of therapeutic lenses, which might overcome some of the barriers to their translation to the clinics, including the use of copolymerisation for enhanced drug-release and preservation of material transparency. Furthermore, until now, 3D (bio)printing has only begun to be explored as a possible production method of contact lenses in dropless drug delivery in ophthalmology. In a recent review by Xu et al. [2], the authors mentioned 3D printing for use in ocular models in experimentation pertaining to drug-eluting contact lenses [116]. In our article, we wanted to emphasise the recent development in this field, enabling the production of custom, patient-specific therapeutic contact lenses, 3D (bio)printing being a smart future choice, especially in the case of multimodal treatment (e.g., controlled drug delivery and cell therapy). Despite a recent review on 3D printing in ophthalmology, in which contact lenses are mentioned in conjunction with drug delivery and diagnostic possibilities [111], the latter possibilities are not mentioned. To our best knowledge, 3D (bio)printing of contact lenses for stem cell therapy was not yet included in any reviews to date. Considering the extensive range of ocular surface pathologies with unmet therapeutic needs (e.g., persistent epithelial defects, limbal epithelial stem cell deficiency in chemical burns and rheumatic pathology, pterygium), which could be transformed with further development of this field, our review might present a very helpful starting point. Figure 14 shows an outlook of the potential use of such an approach in a concrete clinical setting.

## 6. Conclusions

Over the past decade, the field of ocular drug delivery systems has greatly advanced. In search of greater drug bioavailability, contact lens ocular drug delivery has been extensively studied and developed. This article provides an overview of different types of lenses, their properties, and their production, including drug-binding methods. As the properties of different types of lenses vary, so does their compatibility with specific drugs and drug-binding methods, offering a variety of possible clinical applications. However, general clinical application is yet to be seen. Simultaneously, we see an emerging field of 3D printing as a promising customisable method for a personalised treatment approach. The soaking method has the advantage of its simplicity and a higher level of general applicability. However, other methods, such as molecular imprinting and the usage of nanoparticles, have achieved better results concerning drug retention and release control. In order to improve clinical applicability, classic lens properties must be abided by—transparency, oxygen permeability and wettability are among the most important. Further research into the optimisation of these properties in conjunction with materials and drug-binding methods research is needed to ensure real-life applicability, compliance and, ultimately, an improvement of the quality of life of patients.

## Figures and Tables

**Table 1:** A list of colloidal nanoparticles, their examples and characteristics, with relevant references.

Colloidal Nanoparticles	Examples	Characteristics	References
**Polymeric nanoparticles**	PGT nanoparticles EDGMA and PGT (cross-linked nanoparticles) Dispersed bovine serum albumin-coated meloxicam nanocrystals	Favourable release profile Limitation: nanoparticles reduce oxygen and ion permeability in silicone hydrogel CL, as well as increase the storage modulus and decrease the water content	[55,61,62]
**Cyclodextrins**	puerarin-β-CD methacrylated-β-CD GMA-β-CD	Hydrophobic interior can accommodate several hydrophobic molecules, enabling better-controlled drug delivery Increasing tensile strength Inducing swelling	[63,64,65]
**Liposomes**	dimyristoyl phosphatidylcholine liposomes REL ML PEG-biotinylated lipid liposomes	Good biocompatibility and biodegradability, suitable for a variety of medical applications Sustainable drug delivery heavily dependent on the number of lipid barriers Gas permeability compromised due to liposome multilayer	[9,21,33,59,66]
**Microemulsions and micelles**	oil-in-water type microemulsions with ethyl butyrate and Pluronic F127 microemulsion with OTMS silica shell nanostructured microemulsions or micelles MePEG-b-PCL micelles Silica shell nanoparticles	Thermodynamically stable, have a high loading capacity of drugs, while their release profile is readily adaptable Decrease protein adherence and increases wettability of contact lenses Prolonged drug release Hydrogel transparency largely unaffected	[60,67,68,69,70,71]

**Table 2:** Methods of binding drugs into/to lenses. The basics of drug-binding mechanism and methods’ advantages and limitations are described, with relevant references listed.

Method	Mechanism	Advantages	Limitations	References
**Layered contact lenses**	Approaches: The system can make use of different materials, for one of which the drug exhibits tropism, being less diffusible from that region. Alternating layers, which degrade over time.	The layers retard diffusion, ensuring a more stable release.	Compromised gas permeability.	[17,22,33]
**Surface-modified contact lenses**	Loading the drugs into already created polymers or attaching them to the lens surface.	Enables the attachment of drugs to either side of the lens selectively.	Can diminish kinetic friction coefficient.	[21,25]
**Soaking method**	Contact lenses are soaked in the desired drug solution. Some of the drug adheres to the polymer matrix while the rest is dissolved in the aqueous phase before release.	The simplest, cost-effective, and conventional approach. Higher drug bioavailability than eye drops. Some of the drug adheres to the polymer matrix while the rest is dissolved in the aqueous phase before release.	The lens’ ability for drug uptake is highly dependent on the lens’ water content and thickness, the drug’s molecular weight, its concentration in the soaking solution and its solubility in the hydrogel matrix. Lenses show a low affinity for several ophthalmic drugs. Sterilisation and packaging processes might cause a premature release.	[27,28,29,30,33,34,35,36]
**Molecular imprinting**	The targeted drug is mixed with functional monomers. After polymerisation, the drug is removed, and specific cavities in a 3D-polymer network are formed.	Molecular sites with high affinity increase the drug loading capacity while decreasing diffusion rates. Drug affinity and its release profiles can be tailored. Can be used with a variety of monomers (HEMA, MAA, MMA).	The optical and physical properties of the lenses can be altered by the structure of such hydrogels. The gas and ion permeability is dependent on the material’s water content, which decreases with wear time, presenting an obstacle in extended wear uses.	[33,40,41,48,49]
**Colloidal nanoparticles**	The formulated nanoparticular system is dispersed in different monomers (e.g., HEMA) and further polymerised (e.g., with EGDMA) and/or with photo inhibitors.	Can entrap drug molecules and attain better control over their release rate from contact lenses. Prevent the interaction of drugs with the polymerisation mixture and provide additional resistance to the drug’s release.	Alter the lens’s physical and mechanical properties, such as gas and ion permeability, transparency and swelling behaviour.	[33,54,55,56,59,60]
**Supercritical fluid technology**	Supercritical solvents, such as CO_2,_ are used to drugs onto contact lenses. During the impregnation, the dissolution of drugs occurs in a supercritical solvent at conditions that are subcritical or supercritical, followed by an interaction with hydrogels.	Can be used to adjust the drug’s release kinetics without affecting the optical properties of the lens.	Limited loading of drugs with higher solid molar volume (such as dexamethasone) due to constraints in diffusion and uptake by contact lenses.	[33,83,84]
**Vitamin E as a release-modifying additive**	Incorporation of vitamin E into lenses to act as a diffusion barrier.	Slows the drug release rate without significantly altering the lens’s bulk properties. Very stable when incorporated into a lens due to its hydrophobic nature, having negligible solubility in the tear fluid. Can prevent the oxidisation of drugs, such as cysteamine.	Reduces ion permeability and oxygen permeability. Increases storage module due to changes in mechanical properties and protein absorption due to its hydrophobic nature.	[17,33,89,92]

## References

[1] Mitra A.K. (2009). Role of Transporters in Ocular Drug Delivery System. Pharm. Res..

[2] Xu J., Xue Y., Hu G., Lin T., Gou J., Yin T., He H., Zhang Y., Tang X. (2018). A Comprehensive Review on Contact Lens for Ophthalmic Drug Delivery. J Control. Release.

[3] Gote V., Sikder S., Sicotte J., Pal D. (2019). Ocular Drug Delivery: Present Innovations and Future Challenges. J. Pharmacol. Exp. Ther..

[4] Lim L., Lim E.W.L. (2020). Therapeutic Contact Lenses in the Treatment of Corneal and Ocular Surface Diseases—A Review. Asia-Pac. J. Ophthalmol..

[5] Musgrave C.S.A., Fang F. (2019). Contact Lens Materials: A Materials Science Perspective. Materials.

[6] Liechty W.B., Kryscio D.R., Slaughter B.V., Peppas N.A. (2010). Polymers for Drug Delivery Systems. Annu. Rev. Chem. Biomol. Eng..

[7] Aghabegi Moghanjoughi A., Khoshnevis D., Zarrabi A. (2016). A Concise Review on Smart Polymers for Controlled Drug Release. Drug Deliv. Transl. Res..

[8] Stapleton F., Stretton S., Papas E., Skotnitsky C., Sweeney D.F. (2006). Silicone Hydrogel Contact Lenses and the Ocular Surface. Ocul. Surf..

[9] Guzman-Aranguez A., Colligris B., Pintor J. (2013). Contact Lenses: Promising Devices for Ocular Drug Delivery. J. Ocul. Pharmacol. Ther..

[10] Auffarth G.U., Apple D.J. (2001). Zur Entwicklungsgeschichte Der Intraokularlinsen. Der Ophthalmol..

[11] Nicolson P.C., Vogt J. (2001). Soft Contact Lens Polymers: An Evolution. Biomaterials.

[12] Center for Devices and Radiological Health (2020). Class II Daily Wear Contact Lenses—Premarket Notification [510(k)] Guidance Document.

[13] Gonzalez-Chomon C., Concheiro A., Alvarez-Lorenzo C. (2013). Soft Contact Lenses for Controlled Ocular Delivery: 50 Years in the Making. Ther. Deliv..

[14] Mutlu Z., Shams Es-Haghi S., Cakmak M. (2019). Recent Trends in Advanced Contact Lenses. Adv. Health Mater..

[15] Donshik P.C. (2003). Extended Wear Contact Lenses. Ophthalmol. Clin. N. Am..

[16] The Editors of Encyclopaedia Britannica PolyHEMA. https://www.britannica.com/science/polyHEMA.

[17] Dixon P., Shafor C., Gause S., Hsu K.H., Powell K.C., Chauhan A. (2015). Therapeutic Contact Lenses: A Patent Review. Expert Opin. Ther. Pat..

[18] Makadia H.K., Siegel S.J. (2011). Poly Lactic-Co-Glycolic Acid (PLGA) as Biodegradable Controlled Drug Delivery Carrier. Polymer.

[19] Ciolino J.B., Hudson S.P., Mobbs A.N., Hoare T.R., Iwata N.G., Fink G.R., Kohane D.S. (2011). A Prototype Antifungal Contact Lens. Investig. Ophthalmol. Vis. Sci..

[20] Nakada K., Sugiyama A. (1998). Process for Producing Controlled Drug-Release Contact Lens, and Controlled Drug-Release Contact Lens Thereby Produced. U.S. Patent.

[21] Danion A., Brochu H., Martin Y., Vermette P. (2007). Fabrication and Characterization of Contact Lenses Bearing Surface-Immobilized Layers of Intact Liposomes. J. Biomed. Mater. Res. A.

[22] Danion A., Arsenault I., Vermette P. (2007). Antibacterial Activity of Contact Lenses Bearing Surface-Immobilized Layers of Intact Liposomes Loaded with Levofloxacin. J. Pharm. Sci..

[23] Qiu Y. (2017). Silicone Hydrogel Lens with a Crosslinked Hydrophilic Coating. U.S. Patent.

[24] Li J., Zhang Z., Loose C.R., Coury A. (2014). Silicone Hydrogel Contact Lens Modified Using Lanthanide or Transition Metal Oxidants. U.S. Patent.

[25] Korogiannaki M., Samsom M., Schmidt T.A., Sheardown H. (2018). Surface-Functionalized Model Contact Lenses with a Bioinspired Proteoglycan 4 (PRG4)-Grafted Layer. ACS Appl. Mater. Interfaces.

[26] Winterton L.C. (2010). Method of Making Silicone Hydrogel Contact Lenses. U.S. Patent.

[27] Li C.C., Chauhan A. (2007). Ocular Transport Model for Ophthalmic Delivery of Timolol through P-HEMA Contact Lenses. J. Drug Deliv. Sci. Technol..

[28] Kim J., Chauhan A. (2008). Dexamethasone Transport and Ocular Delivery from Poly(Hydroxyethyl Methacrylate) Gels. Int. J. Pharm..

[29] Maulvi F.A., Soni T.G., Shah D.O. (2015). Extended Release of Hyaluronic Acid from Hydrogel Contact Lenses for Dry Eye Syndrome. J. Biomater. Sci. Polym. Ed..

[30] Ruben M., Watkins R. (1975). Pilocarpine Dispensation for the Soft Hydrophilic Contact Lens. Br. J. Ophthalmol..

[31] Soluri A., Hui A., Jones L. (2012). Delivery of Ketotifen Fumarate by Commercial Contact Lens Materials. Optom. Vis. Sci..

[32] Xu J., Li X., Sun F. (2011). In Vitro and in Vivo Evaluation of Ketotifen Fumarate-Loaded Silicone Hydrogel Contact Lenses for Ocular Drug Delivery. Drug Deliv..

[33] Maulvi F.A., Soni T.G., Shah D.O. (2016). A Review on Therapeutic Contact Lenses for Ocular Drug Delivery. Drug Deliv..

[34] Schultz C.L., Poling T.R., Mint J.O. (2009). A Medical Device/Drug Delivery System for Treatment of Glaucoma. Clin. Exp. Optom..

[35] Xinming L., Yingde C., Lloyd A.W., Mikhalovsky S.V., Sandeman S.R., Howel C.A., Liewen L. (2008). Polymeric Hydrogels for Novel Contact Lens-Based Ophthalmic Drug Delivery Systems: A Review. Contact Lens. Anterior Eye.

[36] Maulvi D.F. (2014). Effect of Timolol Maleate Concentration on Uptake and Release from Hydrogel Contact Lenses Using Soaking Method. J. Pharm. Appl. Sci..

[37] Franco P., De Marco I. (2021). Contact Lenses as Ophthalmic Drug Delivery Systems: A Review. Polymers.

[38] Topete A., Oliveira A.S., Fernandes A., Nunes T.G., Serro A.P., Saramago B. (2018). Improving Sustained Drug Delivery from Ophthalmic Lens Materials through the Control of Temperature and Time of Loading. Eur. J. Pharm. Sci..

[39] Minami T., Ishida W., Kishimoto T., Nakajima I., Hino S., Arai R., Matsunaga T., Fukushima A., Yamagami S. (2019). In Vitro and in Vivo Performance of Epinastine Hydrochloride-Releasing Contact Lenses. PLoS ONE.

[40] Zidarič T., Finšgar M., Maver U., Maver T. (2022). Artificial Biomimetic Electrochemical Assemblies. Biosensors.

[41] Alvarez-Lorenzo C., Hiratani H., Gomez-Amoza J.L., Martinez-Pacheco R., Souto C., Concheiro A. (2002). Soft Contact Lenses Capable of Sustained Delivery of Timolol. J. Pharm. Sci..

[42] Hiratani H., Mizutani Y., Alvarez-Lorenzo C. (2005). Controlling Drug Release from Imprinted Hydrogels by Modifying the Characteristics of the Imprinted Cavities. Macromol. Biosci..

[43] Alvarez-Lorenzo C., Yanez F., Barreiro-Iglesias R., Concheiro A. (2006). Imprinted Soft Contact Lenses as Norfloxacin Delivery Systems. J. Control. Release.

[44] Tieppo A., Pate K.M., Byrne M.E. (2012). In Vitro Controlled Release of an Anti-Inflammatory from Daily Disposable Therapeutic Contact Lenses under Physiological Ocular Tear Flow. Eur. J. Pharm. Biopharm..

[45] Venkatesh S., Saha J., Pass S., Byrne M.E. (2008). Transport and Structural Analysis of Molecular Imprinted Hydrogels for Controlled Drug Delivery. Eur. J. Pharm. Biopharm..

[46] Hiratani H., Fujiwara A., Tamiya Y., Mizutani Y., Alvarez-Lorenzo C. (2005). Ocular Release of Timolol from Molecularly Imprinted Soft Contact Lenses. Biomaterials.

[47] White C.J., McBride M.K., Pate K.M., Tieppo A., Byrne M.E. (2011). Extended Release of High Molecular Weight Hydroxypropyl Methylcellulose from Molecularly Imprinted, Extended Wear Silicone Hydrogel Contact Lenses. Biomaterials.

[48] Schrader S., Wedel T., Moll R., Geerling G. (2006). Combination of Serum Eye Drops with Hydrogel Bandage Contact Lenses in the Treatment of Persistent Epithelial Defects. Graefes. Arch. Clin. Exp. Ophthalmol..

[49] Byrne M.E., Salian V. (2008). Molecular Imprinting within Hydrogels II: Progress and Analysis of the Field. Int. J. Pharm..

[50] Tang L., Zhao C.Y., Wang X.H., Li R.S., Yang J.R., Huang Y.P., Liu Z.S. (2015). Macromolecular Crowding of Molecular Imprinting: A Facile Pathway to Produce Drug Delivery Devices for Zero-Order Sustained Release. Int. J. Pharm..

[51] Malakooti N., Alexander C., Alvarez-Lorenzo C. (2015). Imprinted Contact Lenses for Sustained Release of Polymyxin B and Related Antimicrobial Peptides. J. Pharm. Sci..

[52] Alvarez-Rivera F., Concheiro A., Alvarez-Lorenzo C. (2018). Epalrestat-Loaded Silicone Hydrogels as Contact Lenses to Address Diabetic-Eye Complications. Eur. J. Pharm. Biopharm..

[53] Anirudhan T.S., Nair A.S., Parvathy J. (2016). Extended Wear Therapeutic Contact Lens Fabricated from Timolol Imprinted Carboxymethyl Chitosan-g-Hydroxy Ethyl Methacrylate-g-Poly Acrylamide as a Onetime Medication for Glaucoma. Eur. J. Pharm. Biopharm..

[54] Gupta C., Chauhan A. (2010). Drug Transport in HEMA Conjunctival Inserts Containing Precipitated Drug Particles. J. Colloid Interface Sci..

[55] Jung H.J., Abou-Jaoude M., Carbia B.E., Plummer C., Chauhan A. (2013). Glaucoma Therapy by Extended Release of Timolol from Nanoparticle Loaded Silicone-Hydrogel Contact Lenses. J. Control. Release.

[56] Hsu K.H., Gause S., Chauhan A. (2014). Review of Ophthalmic Drug Delivery by Contact Lenses. J. Drug Deliv. Sci. Technol..

[57] Fazly Bazzaz B.S., Khameneh B., Jalili-Behabadi M.M., Malaekeh-Nikouei B., Mohajeri S.A. (2014). Preparation, Characterization and Antimicrobial Study of a Hydrogel (Soft Contact Lens) Material Impregnated with Silver Nanoparticles. Contact Lens. Anterior Eye.

[58] Gulsen D., Chauhan A. (2004). Ophthalmic Drug Delivery through Contact Lenses. Investig. Ophthalmol. Vis. Sci..

[59] Gulsen D., Li C.C., Chauhan A. (2005). Dispersion of DMPC Liposomes in Contact Lenses for Ophthalmic Drug Delivery. Curr. Eye Res..

[60] Gulsen D., Chauhan A. (2005). Dispersion of Microemulsion Drops in HEMA Hydrogel: A Potential Ophthalmic Drug Delivery Vehicle. Int. J. Pharm..

[61] Jung H.J., Chauhan A. (2012). Temperature Sensitive Contact Lenses for Triggered Ophthalmic Drug Delivery. Biomaterials.

[62] Zhang W., Zu D., Chen J., Peng J., Liu Y., Zhang H., Li S., Pan W. (2014). Bovine Serum Albumin-Meloxicam Nanoaggregates Laden Contact Lenses for Ophthalmic Drug Delivery in Treatment of Postcataract Endophthalmitis. Int. J. Pharm..

[63] Loftsson T., Masson M., Brewster M.E. (2004). Self-Association of Cyclodextrins and Cyclodextrin Complexes. J. Pharm. Sci..

[64] dos Santos J.F., Couceiro R., Concheiro A., Torres-Labandeira J.J., Alvarez-Lorenzo C. (2008). Poly(Hydroxyethyl Methacrylate-Co-Methacrylated-Beta-Cyclodextrin) Hydrogels: Synthesis, Cytocompatibility, Mechanical Properties and Drug Loading/Release Properties. Acta Biomater..

[65] dos Santos J.F., Alvarez-Lorenzo C., Silva M., Balsa L., Couceiro J., Torres-Labandeira J.J., Concheiro A. (2009). Soft Contact Lenses Functionalized with Pendant Cyclodextrins for Controlled Drug Delivery. Biomaterials.

[66] Jain R.L., Shastri J.P. (2011). Study of Ocular Drug Delivery System Using Drug-Loaded Liposomes. Int. J. Pharm. Investig..

[67] Chaudhari P., Ghate V.M., Lewis S.A. (2021). Next-Generation Contact Lenses: Towards Bioresponsive Drug Delivery and Smart Technologies in Ocular Therapeutics. Eur. J. Pharm. Biopharm..

[68] Li C.C., Abrahamson M., Kapoor Y., Chauhan A. (2007). Timolol Transport from Microemulsions Trapped in HEMA Gels. J. Colloid Interface Sci..

[69] Kapoor Y., Chauhan A. (2008). Ophthalmic Delivery of Cyclosporine A from Brij-97 Microemulsion and Surfactant-Laden p-HEMA Hydrogels. Int. J. Pharm..

[70] Lu C., Yoganathan R.B., Kociolek M., Allen C. (2013). Hydrogel Containing Silica Shell Cross-Linked Micelles for Ocular Drug Delivery. J. Pharm. Sci..

[71] Maulvi F.A., Mangukiya M.A., Patel P.A., Vaidya R.J., Koli A.R., Ranch K.M., Shah D.O. (2016). Extended Release of Ketotifen from Silica Shell Nanoparticle-Laden Hydrogel Contact Lenses: In Vitro and in Vivo Evaluation. J. Mater. Sci. Mater. Med..

[72] Shayani Rad M., Khameneh B., Sabeti Z., Mohajeri S.A., Fazly Bazzaz B.S. (2016). Antibacterial Activity of Silver Nanoparticle-Loaded Soft Contact Lens Materials: The Effect of Monomer Composition. Curr. Eye Res..

[73] Chandasana H., Prasad Y.D., Chhonker Y.S., Chaitanya T.K., Mishra N.N., Mitra K., Shukla P.K., Bhatta R.S. (2014). Corneal Targeted Nanoparticles for Sustained Natamycin Delivery and Their PK/PD Indices: An Approach to Reduce Dose and Dosing Frequency. Int. J. Pharm..

[74] Xu J., Li X., Sun F. (2010). Cyclodextrin-Containing Hydrogels for Contact Lenses as a Platform for Drug Incorporation and Release. Acta Biomater..

[75] Garcia-Fernandez M.J., Tabary N., Martel B., Cazaux F., Oliva A., Taboada P., Concheiro A., Alvarez-Lorenzo C. (2013). Poly-(Cyclo)Dextrins as Ethoxzolamide Carriers in Ophthalmic Solutions and in Contact Lenses. Carbohydr. Polym..

[76] Glisoni R.J., Garcia-Fernandez M.J., Pino M., Gutkind G., Moglioni A.G., Alvarez-Lorenzo C., Concheiro A., Sosnik A. (2013). Beta-Cyclodextrin Hydrogels for the Ocular Release of Antibacterial Thiosemicarbazones. Carbohydr. Polym..

[77] Arslan M., Gevrek T.N., Sanyal R., Sanyal A. (2015). Fabrication of Poly(Ethylene Glycol)-Based Cyclodextrin Containing Hydrogels via Thiol-Ene Click Reaction. Eur. Polym. J..

[78] Hu X., Tan H., Wang X., Chen P. (2016). Surface Functionalization of Hydrogel by Thiol-Yne Click Chemistry for Drug Delivery. Colloids Surf. Physicochem. Eng. Asp..

[79] Prakash M., Dhesingh R.S. (2017). Nanoparticle Modified Drug Loaded Biodegradable Polymeric Contact Lenses for Sustainable Ocular Drug Delivery. Curr. Drug Deliv..

[80] Danion A., Doillon C.J., Giasson C.J., Djouahra S., Sauvageau P., Paradis R., Vermette P. (2007). Biocompatibility and Light Transmission of Liposomal Lenses. Optom. Vis. Sci..

[81] Bengani L.C., Chauhan A. (2013). Extended Delivery of an Anionic Drug by Contact Lens Loaded with a Cationic Surfactant. Biomaterials.

[82] Kapoor Y., Thomas J.C., Tan G., John V.T., Chauhan A. (2009). Surfactant-Laden Soft Contact Lenses for Extended Delivery of Ophthalmic Drugs. Biomaterials.

[83] Costa V.P., Braga M.E.M., Duarte C.M.M., Alvarez-Lorenzo C., Concheiro A., Gil M.H., de Sousa H.C. (2010). Anti-Glaucoma Drug-Loaded Contact Lenses Prepared Using Supercritical Solvent Impregnation. J. Supercrit. Fluids.

[84] Yanez F., Martikainen L., Braga M.E., Alvarez-Lorenzo C., Concheiro A., Duarte C.M., Gil M.H., de Sousa H.C. (2011). Supercritical Fluid-Assisted Preparation of Imprinted Contact Lenses for Drug Delivery. Acta Biomater..

[85] Zaidi S.A. (2020). Molecular Imprinting: A Useful Approach for Drug Delivery. Mater. Sci. Energy Technol..

[86] Duarte A.R.C., Simplicio A.L., Vega-González A., Subra-Paternault P., Coimbra P., Gil M.H., de Sousa H.C., Duarte C.M.M. (2007). Supercritical Fluid Impregnation of a Biocompatible Polymer for Ophthalmic Drug Delivery. J. Supercrit. Fluids.

[87] Duarte A.R.C., Casimiro T., Aguiar-Ricardo A., Simplício A.L., Duarte C.M.M. (2006). Supercritical Fluid Polymerisation and Impregnation of Molecularly Imprinted Polymers for Drug Delivery. J. Supercrit. Fluids.

[88] Yokozaki Y., Sakabe J., Ng B., Shimoyama Y. (2015). Effect of Temperature, Pressure and Depressurization Rate on Release Profile of Salicylic Acid from Contact Lenses Prepared by Supercritical Carbon Dioxide Impregnation. Chem. Eng. Res. Des..

[89] Peng C.C., Kim J., Chauhan A. (2010). Extended Delivery of Hydrophilic Drugs from Silicone-Hydrogel Contact Lenses Containing Vitamin E Diffusion Barriers. Biomaterials.

[90] Kim J., Peng C.C., Chauhan A. (2010). Extended Release of Dexamethasone from Silicone-Hydrogel Contact Lenses Containing Vitamin E. J. Control. Release.

[91] Peng C.C., Chauhan A. (2011). Extended Cyclosporine Delivery by Silicone-Hydrogel Contact Lenses. J. Control. Release.

[92] Hsu K.H., Fentzke R.C., Chauhan A. (2013). Feasibility of Corneal Drug Delivery of Cysteamine Using Vitamin E Modified Silicone Hydrogel Contact Lenses. Eur. J. Pharm. Biopharm..

[93] Peng C.C., Burke M.T., Chauhan A. (2012). Transport of Topical Anesthetics in Vitamin E Loaded Silicone Hydrogel Contact Lenses. Langmuir.

[94] Peng C.C., Burke M.T., Carbia B.E., Plummer C., Chauhan A. (2012). Extended Drug Delivery by Contact Lenses for Glaucoma Therapy. J. Control. Release.

[95] Hsu K.H., Carbia B.E., Plummer C., Chauhan A. (2015). Dual Drug Delivery from Vitamin E Loaded Contact Lenses for Glaucoma Therapy. Eur. J. Pharm. Biopharm..

[96] Dixon P., Fentzke R.C., Bhattacharya A., Konar A., Hazra S., Chauhan A. (2018). In Vitro Drug Release and in Vivo Safety of Vitamin E and Cysteamine Loaded Contact Lenses. Int. J. Pharm..

[97] Dixon P., Chauhan A. (2019). Carbon Black Tinted Contact Lenses for Reduction of Photophobia in Cystinosis Patients. Curr. Eye Res..

[98] Sekar P., Chauhan A. (2019). Effect of Vitamin-E Integration on Delivery of Prostaglandin Analogs from Therapeutic Lenses. J. Colloid Interface Sci..

[99] Torres-Luna C., Hu N., Tammareddy T., Domszy R., Yang J., Wang N.S., Yang A. (2019). Extended Delivery of Non-Steroidal Anti-Inflammatory Drugs through Contact Lenses Loaded with Vitamin E and Cationic Surfactants. Contact Lens Anterior Eye.

[100] Bron A.J., de Paiva C.S., Chauhan S.K., Bonini S., Gabison E.E., Jain S., Knop E., Markoulli M., Ogawa Y., Perez V. (2017). TFOS DEWS II Pathophysiology Report. Ocul. Surf..

[101] Singh A., Li P., Beachley V., McDonnell P., Elisseeff J.H. (2015). A Hyaluronic Acid-Binding Contact Lens with Enhanced Water Retention. Contact Lens Anterior Eye.

[102] Maldonado-Codina C., Efron N. (2004). Impact of Manufacturing Technology and Material Composition on the Mechanical Properties of Hydrogel Contact Lenses. Ophthalmic. Physiol. Opt..

[103] Maulvi F.A., Singhania S.S., Desai A.R., Shukla M.R., Tannk A.S., Ranch K.M., Vyas B.A., Shah D.O. (2018). Contact Lenses with Dual Drug Delivery for the Treatment of Bacterial Conjunctivitis. Int. J. Pharm..

[104] Sammons W.A. (1989). The Nissel Memorial LectureManufacturing—Materials, Methods and Measurements. J. Br. Contact Lens Assoc..

[105] Anseth K.S., Bowman C.N., Brannon-Peppas L. (1996). Mechanical Properties of Hydrogels and Their Experimental Determination. Biomaterials.

[106] Baker J., Blanch H., Prausnitz J. (1994). Equilibrium Swelling Properties of Weakly Lonizable 2-Hydroxyethyl Methacrylate (HEMA)-Based Hydrogels. J. Appl. Polym. Sci..

[107] Milojević M., Gradišnik L., Stergar J., Skelin Klemen M., Stožer A., Vesenjak M., Dobnik Dubrovski P., Maver T., Mohan T., Stana Kleinschek K. (2019). Development of Multifunctional 3D Printed Bioscaffolds from Polysaccharides and NiCu Nanoparticles and Their Application. Appl. Surf. Sci..

[108] Milojević M., Harih G., Vihar B., Vajda J., Gradišnik L., Zidarič T., Stana Kleinschek K., Maver U., Maver T. (2021). Hybrid 3D Printing of Advanced Hydrogel-Based Wound Dressings with Tailorable Properties. Pharmaceutics.

[109] Štiglic A.D., Gürer F., Lackner F., Bračič D., Winter A., Gradišnik L., Makuc D., Kargl R., Duarte I., Plavec J. (2022). Organic Acid Cross-Linked 3D Printed Cellulose Nanocomposite Bioscaffolds with Controlled Porosity, Mechanical Strength, and Biocompatibility. iScience.

[110] Vajda J., Vihar B., Ćurić L.Č., Maver U., Vesenjak M., Dubrovski P.D., Milojević M. (2023). Sr^2+^ vs. Ca^2+^ as Post-Processing Ionic Crosslinkers: Implications for 3D Bioprinting of Polysaccharide Hydrogels in Tissue Engineering. J. Mater. Res. Technol..

[111] Tan G., Ioannou N., Mathew E., Tagalakis A.D., Lamprou D.A., Yu-Wai-Man C. (2022). 3D Printing in Ophthalmology: From Medical Implants to Personalised Medicine. Int. J. Pharm..

[112] Mohamdeen Y.M.G., Tabriz A.G., Tighsazzadeh M., Nandi U., Khalaj R., Andreadis I., Boateng J.S., Douroumis D. (2022). Development of 3D Printed Drug-Eluting Contact Lenses. J. Pharm. Pharmacol..

[113] Bandari S., Nyavanandi D., Dumpa N., Repka M.A. (2021). Coupling Hot Melt Extrusion and Fused Deposition Modeling: Critical Properties for Successful Performance. Adv. Drug Deliv. Rev..

[114] Alam F., Elsherif M., AlQattan B., Salih A., Lee S.M., Yetisen A.K., Park S., Butt H. (2021). 3D Printed Contact Lenses. ACS Biomater. Sci. Eng..

[115] Gruene M., Pflaum M., Deiwick A., Koch L., Schlie S., Unger C., Wilhelmi M., Haverich A., Chichkov B.N. (2011). Adipogenic Differentiation of Laser-Printed 3D Tissue Grafts Consisting of Human Adipose-Derived Stem Cells. Biofabrication.

[116] Phan C.-M., Bajgrowicz M., Gao H., Subbaraman L.N., Jones L.W. (2016). Release of Fluconazole from Contact Lenses Using a Novel In Vitro Eye Model. Optom. Vis. Sci..

